# Gut microbiome transition across a lifestyle gradient in Himalaya

**DOI:** 10.1371/journal.pbio.2005396

**Published:** 2018-11-15

**Authors:** Aashish R. Jha, Emily R. Davenport, Yoshina Gautam, Dinesh Bhandari, Sarmila Tandukar, Katharine M. Ng, Gabriela K. Fragiadakis, Susan Holmes, Guru Prasad Gautam, Jeff Leach, Jeevan Bahadur Sherchand, Carlos D. Bustamante, Justin L. Sonnenburg

**Affiliations:** 1 Department of Biomedical Data Science, Stanford University, Stanford, California, United States of America; 2 Center for Computational, Evolutionary, and Human Genetics, Stanford University, Stanford, California, United States of America; 3 Department of Molecular Biology and Genetics, Cornell University, Ithaca, New York, United States of America; 4 Public Health Research Laboratory, Institute of Medicine, Maharajgunj, Kathmandu, Nepal; 5 Department of Microbiology and Immunology, Stanford University, Stanford, California, United States of America; 6 Department of Statistics, Stanford University, Stanford, California, United States of America; 7 Department of Geography, Tribhuvan University, Nepalgunj, Nepal; 8 Human Food Project, Terlingua, Texas, United States of America; 9 Department of Twin Research and Genetic Epidemiology, King’s College London, St. Thomas’ Hospital, London, United Kingdom; 10 Chan Zuckerberg Biohub, San Francisco, California, United States of America; 11 Center for Human Microbiome Studies, Stanford University, Stanford, California, United States of America; Colorado State University, United States of America

## Abstract

The composition of the gut microbiome in industrialized populations differs from those living traditional lifestyles. However, it has been difficult to separate the contributions of human genetic and geographic factors from lifestyle. Whether shifts away from the foraging lifestyle that characterize much of humanity’s past influence the gut microbiome, and to what degree, remains unclear. Here, we characterize the stool bacterial composition of four Himalayan populations to investigate how the gut community changes in response to shifts in traditional human lifestyles. These groups led seminomadic hunting–gathering lifestyles until transitioning to varying levels of agricultural dependence upon farming. The Tharu began farming 250–300 years ago, the Raute and Raji transitioned 30–40 years ago, and the Chepang retain many aspects of a foraging lifestyle. We assess the contributions of dietary and environmental factors on their gut-associated microbes and find that differences in the lifestyles of Himalayan foragers and farmers are strongly correlated with microbial community variation. Furthermore, the gut microbiomes of all four traditional Himalayan populations are distinct from that of the Americans, indicating that industrialization may further exacerbate differences in the gut community. The Chepang foragers harbor an elevated abundance of taxa associated with foragers around the world. Conversely, the gut microbiomes of the populations that have transitioned to farming are more similar to those of Americans, with agricultural dependence and several associated lifestyle and environmental factors correlating with the extent of microbiome divergence from the foraging population. The gut microbiomes of Raute and Raji reveal an intermediate state between the Chepang and Tharu, indicating that divergence from a stereotypical foraging microbiome can occur within a single generation. Our results also show that environmental factors such as drinking water source and solid cooking fuel are significantly associated with the gut microbiome. Despite the pronounced differences in gut bacterial composition across populations, we found little differences in alpha diversity across lifestyles. These findings in genetically similar populations living in the same geographical region establish the key role of lifestyle in determining human gut microbiome composition and point to the next challenging steps of determining how large-scale gut microbiome reconfiguration impacts human biology.

## Author summary

Although much of humanity’s history has been spent foraging in the forests, the advent of agriculture approximately 10,000 years ago and industrialization approximately 250 years ago mark major shifts in human lifestyle. Several studies have investigated the effect of industrialization on the human gut microbiome—a collection of microbes that inhabit the human gut. However, little is known about whether the gut microbiome changed as humans shifted away from foraging. To investigate how the gut community changes in response to shifts in traditional human lifestyles, we characterized the gut microbial community from four Himalayan populations representing diverse subsistence strategies. We show that the divergence of the gut microbiome from the foraging population is strongly correlated with agricultural dependence in these populations. Many of the taxa that differ across lifestyles are known to be influenced by diet, but we also demonstrate that environmental factors, such as sources of drinking water, are strongly associated with the human gut microbiome. Our findings show that both diet and environment play key roles in shaping the human gut microbiome.

## Introduction

The human gut is comprised of a diverse community of bacteria, the microbiome or microbiota, that influences several aspects of human physiology, including nutrient metabolism, immune responses, and resistance to infectious pathogens [1–3]. This highly malleable microbial component of human biology exhibits rapid, and in some cases, irreversible changes in response to dietary and environmental factors [4–11]. Modern humans have experienced diverse environments since expanding out of Africa approximately 100,000 years ago. Over the past approximately 10,000 years, hunting and gathering has largely yielded to different forms of agriculturally supported lifestyles. More recently, the diet of billions of people has undergone profound changes with the advent of industrialism. Dietary changes combined with a variety of other factors associated with the industrial revolution have been credited as contributing to the alterations in the gut microbiome in industrialized populations [12]. However, interpretation of the current data is clouded by potential contributions of human genetic variation, environment, and geographical factors [5,7,13]. While current evidence is consistent with the extent of lifestyle change impacting the gut microbiome [14], to what extent shifts in lifestyles away from a foraging lifestyle influence gut microbiomes remains poorly understood. Moreover, whether shifts in lifestyles influence gut microbiomes in preindustrial populations remains poorly understood.

Previous studies of the gut microbiome have demonstrated a stark contrast between industrialized versus unindustrialized populations. Comparisons of the gut microbiomes of traditional human populations in Africa and South America with those of the industrialized Western populations from Europe and the United States of America reveal that the human gut microbiome varies across geography and corresponds to differences in lifestyles [15–30]. Microbiome differences between these populations are often large and stark. However, since these populations reside in geographically distinct regions, represent extreme modes of human subsistence, and are culturally distinct, identifying the factors responsible for microbiome differences remains a challenge, as diet, sanitation, and access to medical care are often associated with geographic and cultural features that differentiate populations being compared and confound lifestyle variables. For example, one common trend from these studies is the higher diversity of gut bacteria in unindustrialized traditional populations. However, comparison of human populations that reside in close geographical proximities but practice different types of subsistence have shown little differences in alpha diversity across lifestyles [19,22,29]. Most of the traditional societies investigated thus far live within tropical latitudes, which differ from other regions of the world in macroecological biodiversity, climate, and numerous other factors. Hence, whether difference in alpha diversity between these traditional societies and Europeans is due to contrasting lifestyles, residence in the tropics, or other factors remains unclear [31]. Additionally, it remains unknown when microbiome compositions shifted during the process of industrialization and how long it took for those transitions to occur. Hence, understanding how transitions in human lifestyles lead to changes in the gut microbiomes would be greatly aided by studying populations that cohabit similar geographic regions and have undergone recent changes in culture, lifestyle, and diet.

In order to explore how a gradient of traditional lifestyles may affect the human gut microbiome, we have analyzed the gut microbiomes from four rural Himalayan populations. The Himalayan populations include the Chepang (a foraging population), the Raute and Raji (two foraging communities that are currently transitioning to subsistence farming), and the Tharu (former foragers that have completely transitioned to farming within the last two centuries). We assessed contributions of lifestyle, diet, and environment on the gut microbial variation in the rural Himalayan populations. To further assess how the gut microbiomes of these traditional groups differ from an industrial population, we compared them to Americans with European ancestry. Our results show that gut microbiome composition mirrors the transitions from a traditional to an agrarian lifestyle in Himalaya. In addition to the dietary gradient across these populations, intra- and interpopulation variability in lifestyle elucidated additional environmental factors that may contribute to microbiota change.

## Results

### Description of populations

**Fig 1:**
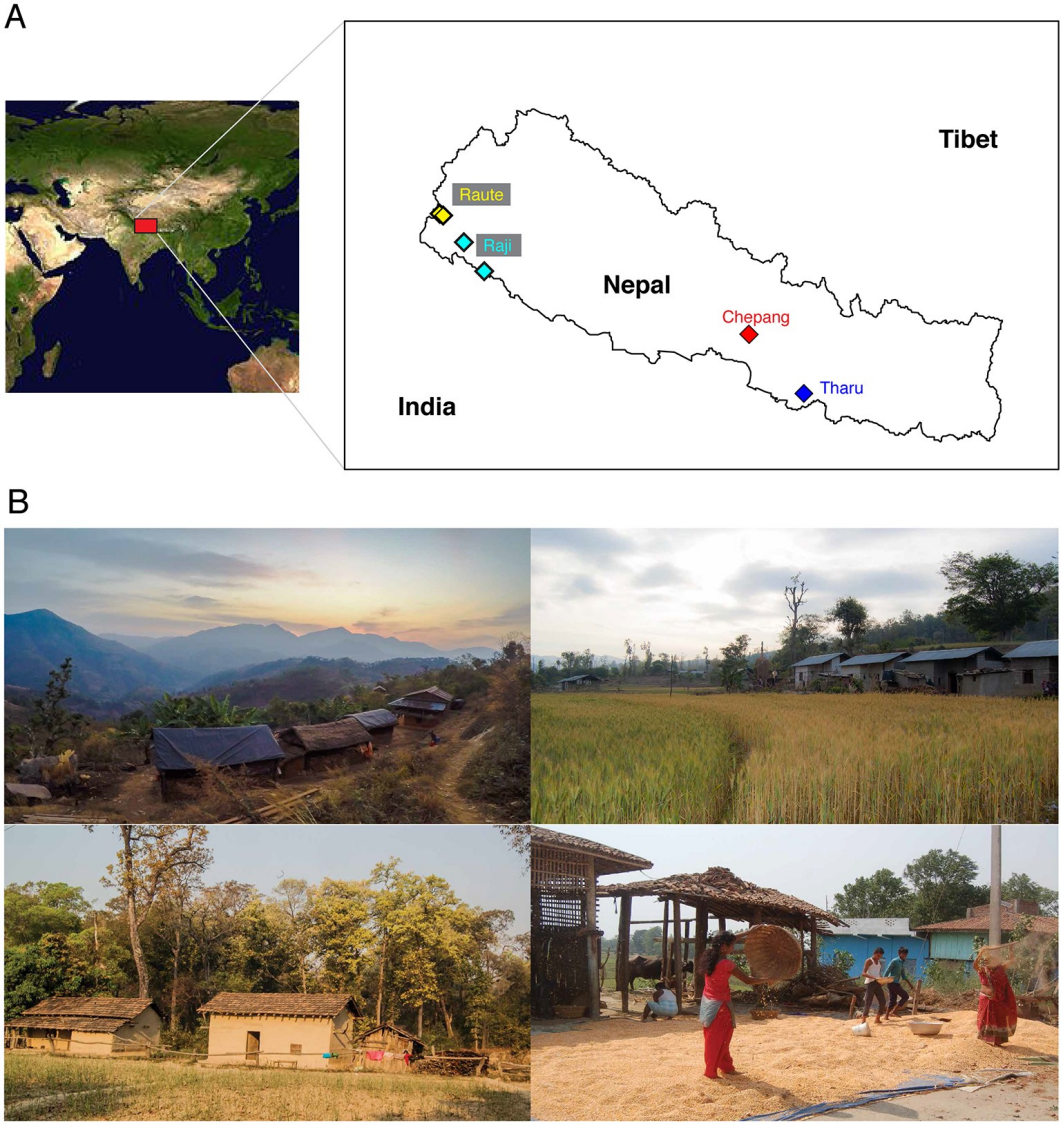
Sampling locations and habitats of the Himalayan populations in Nepal. (A) Map displaying the geographical locations of sampled villages in southern Nepal (altitudes <1,000 m above sea level, latitude 26.97–29.15 °N). The Tharu are geographically most distant from the Raute and Raji and reside closer to the Chepang. (B) Habitats of each population. From top left in a clockwise direction, the remote Chepang village, the Raute village, the Tharu harvesting rice, and the Raji village. The census population sizes of the Raute, Raj, Chepang, and Tharu are 650, 3,758, 48,476, and 1.5 million, respectively.

Our participants included 54 individuals from four Himalayan groups, including Chepang (*N* = 14), Raji (*N* = 9), Raute (*N* = 11), and Tharu (*N* = 20), with median age of 40 years (SD ± 14 years) from rural villages in Nepal (Fig 1 and S1 Table). These four populations are long-term residents of the Himalayan foothills (altitude less than 1,000 m). Ethnographic, linguistic, and cultural data suggest that these populations are of East Asian ancestries, they speak closely related languages, and their cultural practices are similar to one another [32–34]. Although all four of the Himalayan populations in this study were foragers until recently [35–38], habitat loss due to rapid deforestation, population expansions of non-native groups, establishment of new settlements, and construction of modern highways led to settlement of these groups at various time points in the last 300 years. Historical records indicate that the Tharu gradually transitioned into agrarian lifestyles beginning in the late 18th century (250–300 years ago) [38]. They have fully transitioned into farming and are virtually completely disengaged from foraging practices. The population size of the Tharu is approximately 1.5 million, and they are distributed throughout the Terai plains in Nepal [39]. Historically, the Raute, Raji, and Chepang were seminomadic foragers, and their diets included native tubers, greens, and fruits from the jungle and wild honey, fish, and occasional game [36,37,40]. The Raute and Raji abandoned their foraging lifestyles in the 1980s [35,36]. While the Raute have settled in the remote hills in western Nepal, the Raji have settled in the Terai plains, which are relatively more urbanized. The current census size of Raute and Raji are approximately 650 and approximately 3,750, respectively [39]. The Chepang were fully nomadic at least until 1848 [41] and began supplementing their foraging practices with subsistence agriculture less than a century ago [37]. The Chepang population size is approximately 48,500 [39]; however, they exist as fragmented tribes in small, geographically isolated villages of a few hundred individuals deep within the hills of lower Himalaya. The Chepang in this study currently inhabit a remote village that is devoid of modernity, with no electricity, running water, irrigation, fertilizers, modern machines, or marketplaces. They still practice slash and burn agriculture and are completely dependent on rainwater for farming. Because yields from such traditional farming are low, their daily diet consists of wild plants such as *sisnu* (nettles) that are foraged from the forests.

### Lifestyle gradients in the Himalayan populations

We conducted surveys to assess the extent of lifestyle change as these seminomadic populations transitioned to farming in the last few hundred years. The survey questionnaire included questions pertaining to current dietary practices, traditional and modern medicines, and several environmental factors, including sources of drinking water, types of cooking fuel, alcohol use, and tobacco consumption (*N* = 53, S2 Table). We also surveyed presence of parasites in our participants microscopically.

**Fig 2:**
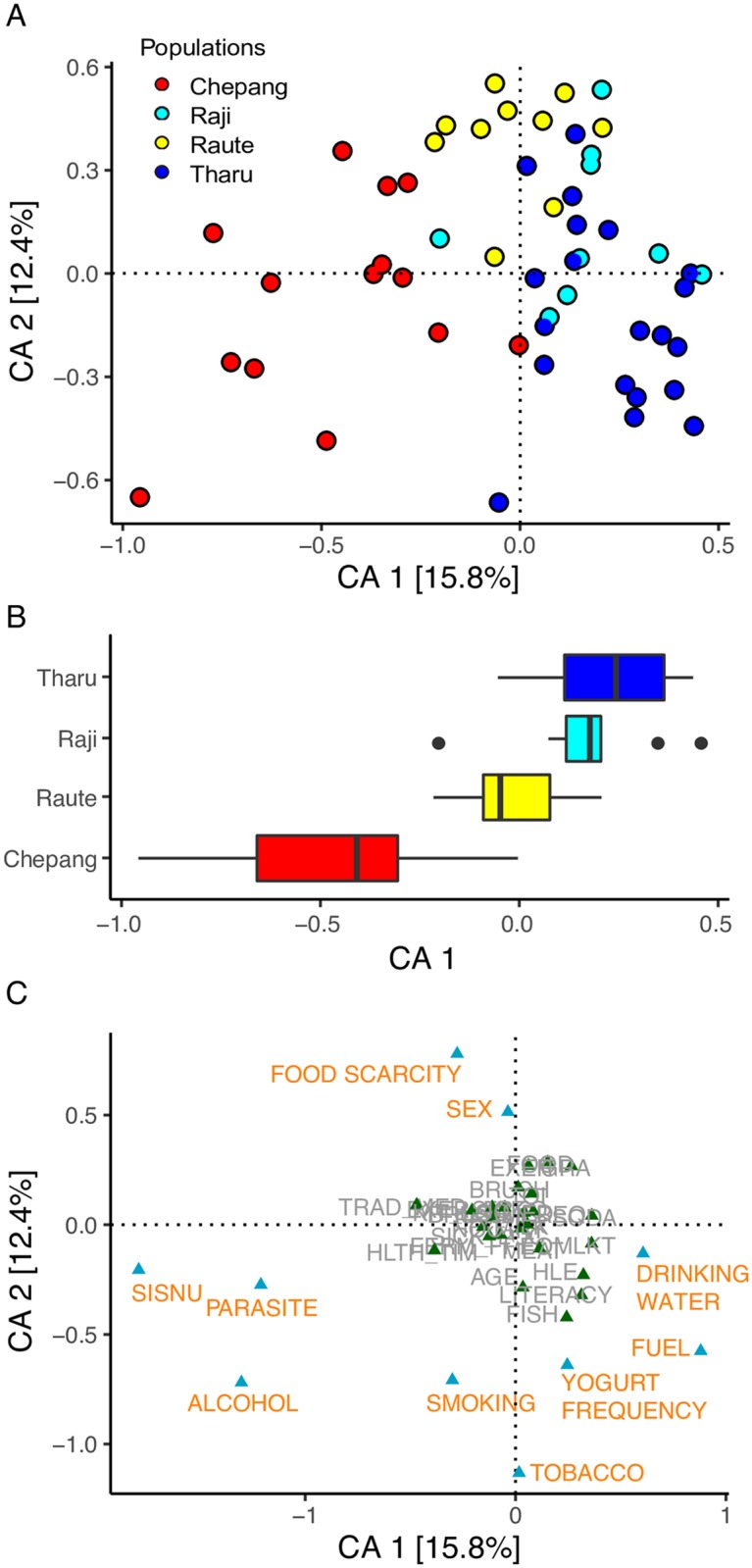
CA based on survey questionnaires and parasite assessment in the Himalayan populations. (A) First two dimensions of the CA and the amount of variation explained are shown. Each circle represents an individual, and colors represent the populations. (B) Distribution of populations along the primary CA1 axis shows patterns of separation by lifestyles. Chepang foragers (red) and Tharu farmers (blue) are on two extreme ends of CA1. In between the two are the Raute (yellow) and Raji (cyan), the two communities that are transitioning from foraging to farming. (C) Factors in gold are those that have more than expected eigenvalues and thus contribute most to the top two dimensions in the CA. The data underlying this figure can be found in S4 Table. CA, correspondence analysis.

Supervised learning using a Random Forest classifier model on the survey data (including intestinal parasite load) assigned the individuals to their respective populations with high overall accuracy (94%, AUC = 0.997, S1 Fig). The Chepang, Raute, and Tharu were classified with 100% accuracies, indicating these populations have distinct lifestyles (S3 Table). 67% of the Raji individuals were classified accurately as Raji while the remainders were classified as Tharu. A correspondence analysis (CA) of the survey data (including intestinal parasite load) also revealed lifestyle differences between these populations (Fig 2A). The first CA dimension (CA1) explained 15.8% variation in the data and was strongly correlated with lifestyle gradients. Along CA1, samples progressed from the Chepang foragers at one extreme, to the Raute and Raji transitioning populations, and then to the Tharu farmers at the opposite extreme (Fig 2B). Despite the geographical distance between them, the Raji lifestyle appears to be more similar to that of the Tharu farmers, consistent with the Raji settlement occurring in a more urbanized setting compared to the Raute. Similarly, the Raute reside in geographical proximity to the Raji, although their lifestyle partitions between the Raji and the Chepang, indicating geographical proximity is not driving the lifestyle differences.

A total of 10 variables contributed highly to the first two CA dimensions, and most of them are strongly associated with dietary differences and modernity (Fig 2C). These differences are described in detail in S2 Fig. Briefly, foraged plants such as *sisnu* (nettles) and *jaand*, a slushy alcoholic beverage made from fermenting millet or corn, are staples of the Chepang diet. In contrast, *sisnu* and *jaand* consumption was minimal among the Raute, Raji, and Tharu. Also, perceived food scarcity was higher in the Chepang and Raute relative to the Raji and Tharu. Although meat consumption was low across all four populations, the Tharu consumed animal products such as yogurt more frequently than the other three populations. Furthermore, the Tharu and Raji also showed increased signs of modernity. For example, they have installed tube wells at their homes, enabling access to underground water for drinking. In contrast, the Chepang and Raute still fetch drinking water from rivers and streams. Also, use of solid biomass fuel was lower in the Tharu and Raji, while the Chepang and Raute are still completely dependent on burning firewood for cooking. Although we detected low overall levels of intestinal parasites across the participants, *Ascaris*, *Entamoeba*, *Trichuris*, *Hymenolepis*, and *Coccidia* were detected in some, and most of the infected were the Chepang. Together, the diet and lifestyle assessments provide unbiased support that the four populations represent a gradient from traditional to increasingly agrarian and urban lifestyles.

### Gut microbiome composition varies by lifestyles

In order to assess whether the gut microbiome varies across lifestyles, we characterized the gut bacterial composition of these populations using the Illumina MiSeq to sequence the V4 region of 16S ribosomal RNA (rRNA) gene obtained from a total of 79 stool samples (including technical replicates), with an average of 11,570 (±4,653) high-quality reads per sample (S3 Fig and S4 Table). Since flash freezing of the samples was not possible in the remote sampling areas in the Himalaya, we used commercially available DNAGenotek OMNIgene kits to collect stool samples from the four populations (*N* = 54). We also collected stool samples from 10 Americans of European descent using OMNIgene kits and compared them with freshly frozen samples to evaluate whether the preservation method affected the microbiome profile. The 16S rRNA profiles of the same samples stored by flash freezing or by OMNIgene were remarkably similar, with reproducible differences in minor taxa (Euryarcheota and Cyanobacteria), demonstrating the reliable preservation of microbiome composition with the OMNIgene kits (S4 Fig). Due to the reproducible, albeit minor, differences between the two collection methods, we used the OMNIgene data from the Americans for consistency in subsequent comparative analyses. The American samples provide a thoroughly investigated population as an industrialized reference for the Himalayan data.

Comparison of the community structure in the five study populations using unweighted UniFrac distances, a measure of compositional similarity that includes the phylogenetic relatedness between microbiomes, showed that the gut microbial composition varies across populations (*P* < 2.2 × 10^−16^, Kruskal–Wallis test, S5 Table). Within Himalaya, the Chepang foragers were closest to the Raute, and the distance between the two was significantly smaller than the Chepang–Tharu distance and marginally smaller than the Chepang–Raji distance (false discovery rate [FDR] adjusted *P* = 5.7 × 10^−4^ and 0.057, respectively; Dunn’s posthoc test). The Raute, Raji, and Tharu were equidistant from one another (FDR adjusted *P* = 0.99 for all pairwise comparisons, Dunn’s posthoc test). Similar results were also observed with weighted UniFrac and Bray–Curtis distances, both of which take the taxa abundance into account (S5 Table). These results suggest that differences in traditional lifestyles as these groups transition from foraging to farming influence their gut microbiomes. Comparison of these traditional Himalayan populations with industrialized Americans showed that all four Himalayan populations exhibited much larger distances from the Americans than when compared to one another (*P* < 1.3 × 10^−5^ for all pairwise comparisons, Dunn’s posthoc test, S5 Table). The Chepang were the most distant from the Americans, followed by the Raute, while the Raji and Tharu were equally close to the Americans.

**Fig 3:**
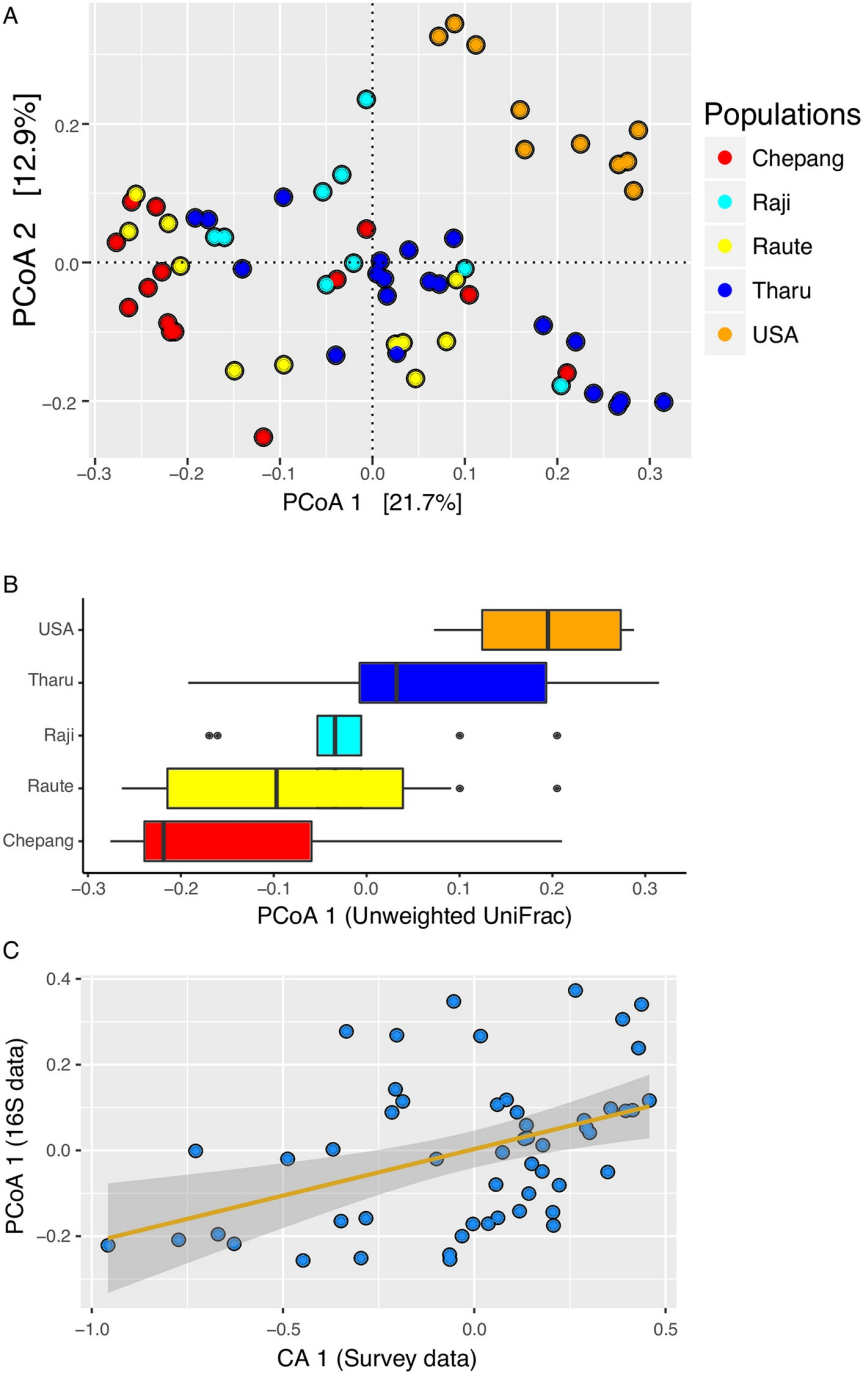
Gut microbiome compositions show gradients corresponding to lifestyles. (A) PCoA of the unweighted UniFrac distances of the 16S rRNA data colored by populations. Each dot represents an individual, and colors indicate the populations. Chepang foragers (red), Raute (yellow), and Raji (cyan) communities that are transitioning from foraging to farming; Tharu farmers (blue); and Americans (orange). (B) Distributions of populations along the PCoA1 axis show patterns of separation by lifestyles. (C) Gut microbial composition of the Himalayan populations represented by the primary dimension of the unweighted UniFrac distance (PCoA1) strongly correlates with lifestyle differences represented by the top dimension of the corresponding analysis performed on the survey data (CA1, Spearman’s Rho = 0.44 and *P* value = 0.001). Correlation between CA2 and PCoA1 was not statistically significant. The data underlying this figure can be found in S1 Data. CA, correspondence analysis; PCoA, Principal Coordinates Analysis; rRNA, ribosomal RNA.

Visualization of these distances using a Principal Coordinates Analysis (PCoA) revealed separation of populations along the top two dimensions (*P* = 1 × 10^−5^, permutational multivariate analysis of variance [PERMANOVA], Fig 3A). Furthermore, gradients in lifestyles were reflected by the distribution of populations along the primary axis (PCoA1, Fig 3B). These distributions remained consistent when using Bray–Curtis and weighted UniFrac distances as well (*P* = 1 × 10^−5^ for both, PERMANOVA, S5 and S6 Figs).

When American microbiomes were eliminated from the PCoAs, the gradient between the Himalayan populations remained pronounced (*P* = 1 × 10^−5^, PERMANOVA, S7 Fig). Among the four Himalayan populations, the strongest separation was observed between the Chepang foragers and the Tharu farmers.

A random forest classifier based on the 16S rRNA-defined amplicon sequence variant (16S ASV) data assigned the Chepang, Tharu, and American individuals to their respective source populations with 79%, 100%, and 100% accuracies (overall accuracy = 66%, AUC = 0.9, S8 Fig and S3 Table). The classification accuracy for the Raute and Raji, the two populations that recently transitioned from foraging to farming, were relatively poor (<10%). While some of the individuals from these groups were classified as the Chepang, others were classified as the Tharu. However, none of the Himalayan individuals were classified as American. These results show that the gut microbiome of the Chepang foragers differs from that of the Tharu farmers, while that of the Raute and Raji reflect a transitional state in their lifestyles. They also indicate that the gut microbiome compositions of the Himalayan populations are distinct from those of the Americans. Therefore, these findings collectively indicate that the transition from foraging to farming is accompanied by noticeable shifts in gut microbiome, which may be further exacerbated in industrial populations.

To formally evaluate whether variation in gut microbiota reflects lifestyle differences within Himalaya, we assessed associations between the respective primary dimensions from the lifestyle questionnaire, parasite analysis (CA1), and gut microbial composition analysis (PCoA1 calculated using the four Himalayan populations) (Fig 3C and S5 Fig). We found that the CA1 was strongly correlated with the PCoA1 obtained from all of the three distance matrices (Spearman’s Rho = 0.47, 0.44, and 0.28 for Bray–Curtis; unweighted UniFrac; and weighted UniFrac distances, respectively; *P* values = 4.5 × 10^−4^, 1.1 × 10^−3^, and 0.05; correlation test). The CA1 was also correlated with PCoA2 of all three distance matrices (Spearman’s Rho = 0.26, 0.44, and 0.39; *P* values = 0.06, 0.001, and 0.004 for Bray–Curtis; unweighted UniFrac; and weighted UniFrac distances; correlation test). Conversely, no significant correlations were detected between CA2 and either of the PCoA axes from all three distances (*P* value > 0.05, correlation test). Notably, CA1 but not CA2 is associated with lifestyle gradient in Himalaya (Fig 2). Strong and consistent correlations between CA1 and PCoA axes indicate that gut microbiome compositions of the Himalayan populations mirror their lifestyles.

### Gut bacterial diversity (alpha diversity) does not vary across lifestyles

**Fig 4:**
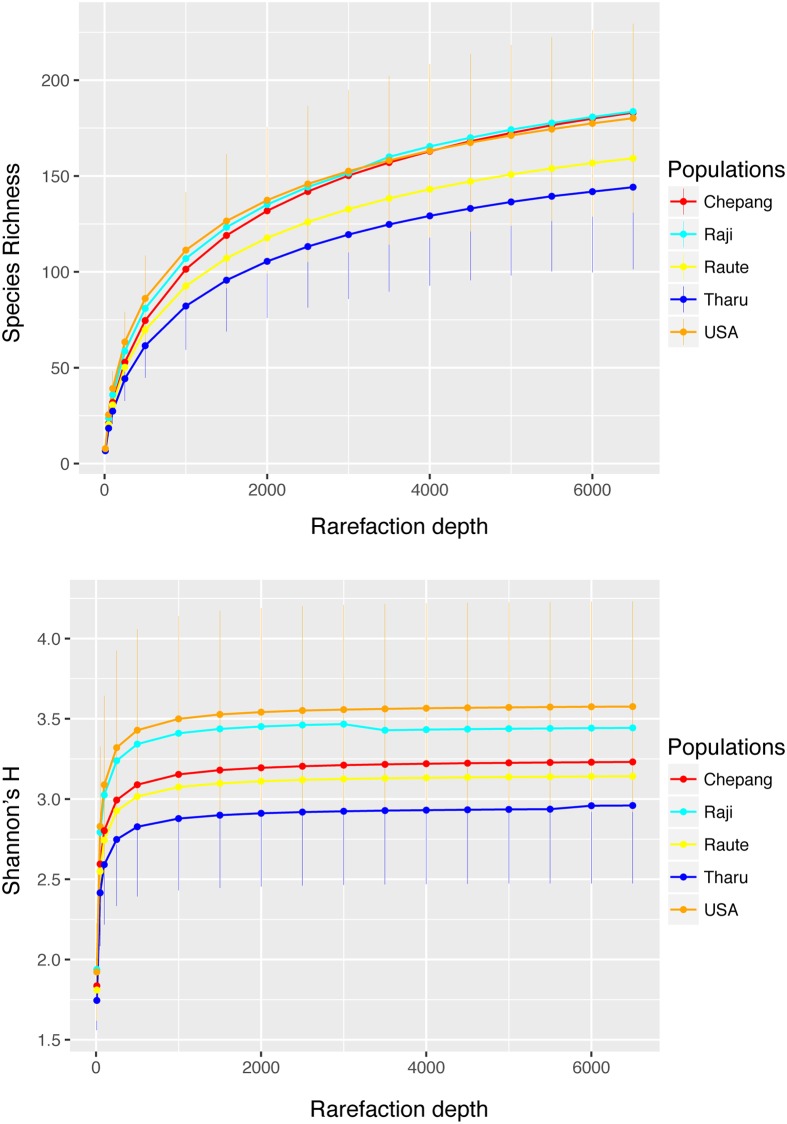
Alpha diversity across lifestyles. Rarefaction curves showing two commonly used measures of alpha diversity—species richness (top) and Shannon’s H (bottom) calculated by subsampling 10–6,500 reads per sample. No significant differences in species richness was detected between the five study populations at a lower depth of 3,000 reads per sample, which included all 64 samples, or at 6,500 reads per sample, which included 61 samples. Shannon’s H was significantly lower in the Tharu relative to the Americans at both rarefaction depths. No differences in any of these two alpha diversity metrics were observed between the Chepang, Raji, Raute, and the Americans. Population labels are colored to indicate the range of different lifestyles (red, foragers; yellow and cyan, former foragers; blue, farmers; orange, industrialists). The data underlying this figure can be found in S1 Data.

Previous studies have suggested that elevated species diversity in the gut microbiome is a hallmark of traditional populations [19,24]. We assessed the alpha diversity in the five study populations using species richness and Shannon’s H at various rarefaction depths ranging from 10–6,500 reads (Fig 4). Species richness measures the presence and absence of taxa, whereas Shannon’s H additionally accounts for the relative abundances of each taxon within each population. We compared alpha diversity across the five populations at a rarefaction depth of 3,000 to include all 64 samples and at a higher rarefaction depth of 6,500, which included 61 samples. Regardless of the rarefaction depth, species richness was not significantly different between any of the five populations (*P* > 0.05, Kruskal–Wallis test). We did find marginally significant differences in Shannon’s H between these populations (*P* = 0.01 and 0.03 at 3,000 and 6,500 rarefaction depths, Kruskal–Wallis test). A posthoc pairwise comparison of all five populations showed that only the alpha diversity in the Tharu was slightly lower than that in the Americans (FDR adjusted *P* = 0.02 and 0.045 at the two rarefaction depths, respectively; Dunn’s posthoc test). Next, we evaluated association between the 10 factors that differentiate the lifestyle of the Himalayan populations (Fig 2) with the two alpha diversity measures. Neither species richness nor Shannon’s H were significantly associated with any of these factors at either rarefaction depth (*P* > 0.05, nonlinear mixed effects model). Finally, we assessed additional metrics of diversity (Fisher’s alpha, Simpson’s D), which similarly fail to differentiate populations (S9 Fig). These results indicate that lifestyle differences among the Himalayan populations or between these populations and Americans have little effect on the alpha diversity of their gut microbiome.

### Bacterial taxa are associated with lifestyle transitions

**Fig 5:**
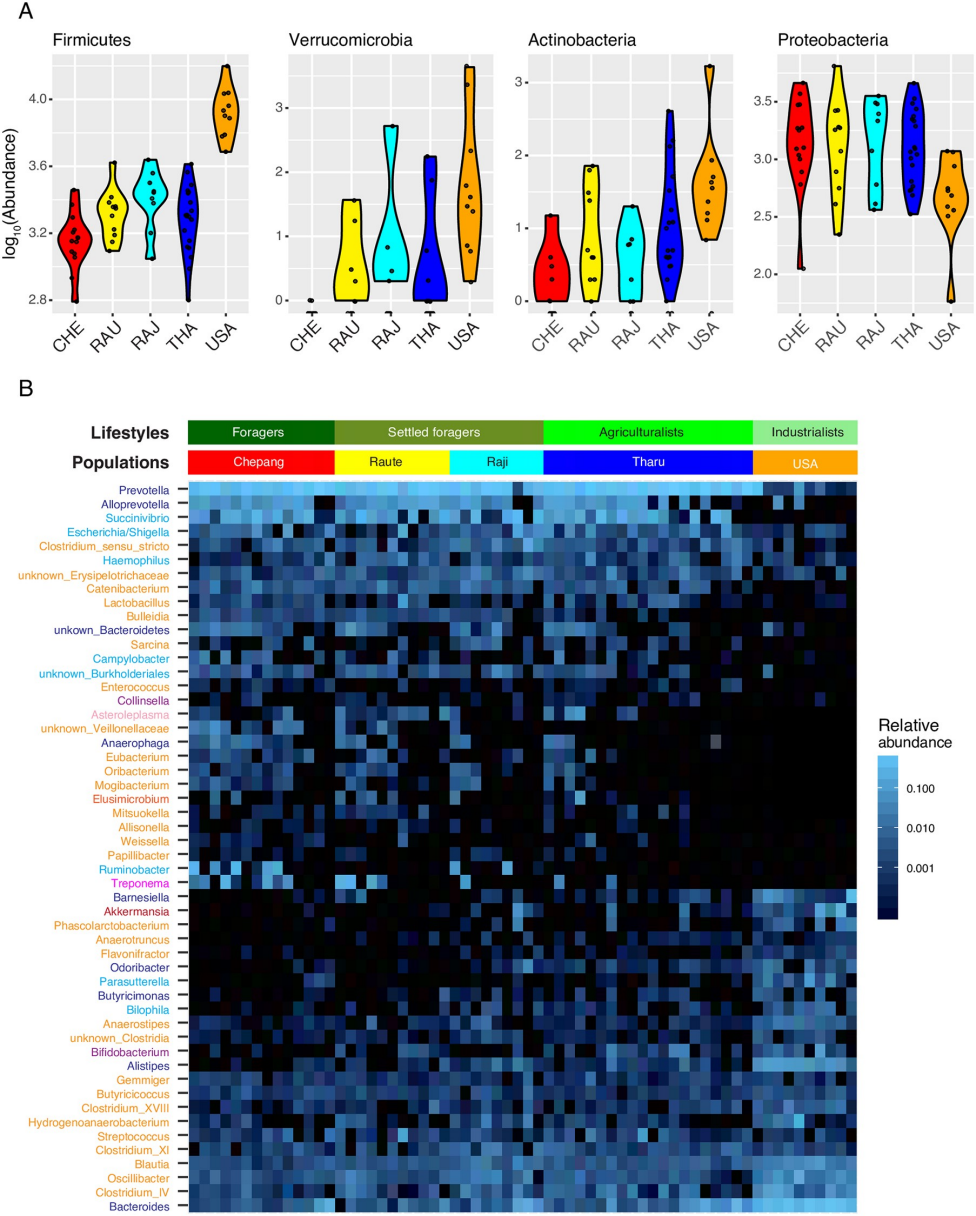
Distinctions in the gut microbiome across lifestyles. (A) Phyla with most significant differences in abundances between the five populations. Abundances of Firmicutes, Verrucomicrobia, and Actinobacteria reflect gradients of traditional industrialized lifestyles. Proteobacteria distinguishes rural Himalayan populations from the Americans. (B) Heatmap displaying 52 genera with significantly different abundance across the five populations. Bars on the top represent the grouping of individuals in the heatmap columns by their populations or lifestyles. Genera labels in rows are colored by their phylum. Purple, Actinobacteria; dark blue, Bacteroidetes; light red, Elusimicrobia; orange, Firmicutes; light blue, Proteobacteria; magenta, Spirochaetes; light pink, Tenericutes; brown, Verrucomicrobia. Heatmap colors reflect relative abundances of each genus. Among the Himalayan populations, *Ruminobacte*r, *Campylobacter*, unknown Veillonellaceae genus, *Bulleidia*, *Weissella*, *Treponema*, *Barnesiella*, *Odoribacter*, *Alistipes*, and *Bifidobacterium* differed significantly. The data underlying this figure can be found in S6 and S7 Tables.

Although lifestyle differences have little effect on the alpha diversity, gut microbiome compositions of the Himalayan populations reflected the gradient in their lifestyles. To identify taxa driving the differences in the gut microbiomes across lifestyles, we compared the abundance of individual phyla across the five populations using a negative binomial generalized linear model (GLM), as implemented in differential expression analysis for sequence count data version 2 (DESeq2) [42]. Differential abundances were detected for six out of 10 phyla (FDR adjusted *P* values are shown in S6 Table), and four of the six phyla reflect a traditional western lifestyle gradient. The Himalayan populations were characterized by higher abundance of Proteobacteria, while abundances of Actinobacteria, Firmicutes, and Verrucomicrobia were highest in the Americans, intermediate in the farmers (Tharu, Raji, and Raute), and lowest in the Chepang foragers (Fig 5A). Higher levels of Proteobacteria and lower levels of Actinobacteria and Verrucomicrobia are common features of many traditional human gut microbiomes around the world [19,21,24,29].

To characterize the taxonomic differences between populations at a finer taxonomic level, we repeated the above analysis at the genus level and identified 52 out of 116 genera that showed significant differences in abundance across the five populations (Fig 5B, FDR adjusted *P* values are shown in S7 Table). The majority of these genera show consistent differences along the lifestyle gradient within the Himalayan samples (Fig 5B). For example, among the Himalayan populations, the Chepang foragers were enriched for *Ruminobacter*, *Campylobacter*, and *Treponema* relative to the Tharu farmers (S10 Fig). Although we did not detect significant differences in the abundance of Bacteroidetes phylum across these populations, several members of this phylum distinguished the Himalayan and American populations. The rural Himalayan communities were enriched for *Prevotella*, *Alloprevotella*, and *Anaerophaga* and significantly depleted in *Bacteroides*, *Alistipes*, *Butyricimonas*, *Odoribacter*, and *Barnesiella*. 29 genera belonging to Firmicutes differed significantly across the five populations, and their distribution was complex across these populations (S11 Fig). The Himalayan populations were enriched for *Clostridium sensu stricto*, *Catenibacterium*, *Lactobacillus*, *Bulleidia*, *Sarcina*, *Enterococcus*, *Eubacterium*, *Oribacterium*, *Mogibacterium*, *Mitsuokella*, *Allisonella*, *Weissella*, *Papilbacter*, and two unknown genera of Erysipelotrichaceae and Veillonellaceae families. Alternatively, abundances of several *Clostridium* genera, *Oscillibacter*, *Blautia*, *Butyriciococcus*, *Anaerostipes*, and *Flavonifractor* were elevated in the Americans. The Americans also showed highest abundances of *Bifidobacterium* (Actinobacteria) and *Akkermansia* (Verrucomicrobia), both of which were extremely low in the Chepang foragers and intermediate in the Tharu farmers. Elevated abundances of *Treponema* and *Prevotella* with reduction of *Bacteroides* and *Bifidobacterium* is a characteristic feature of gut microbiomes of foraging communities [19,21,24,29].

### Microbiome structure across lifestyles

**Fig 6:**
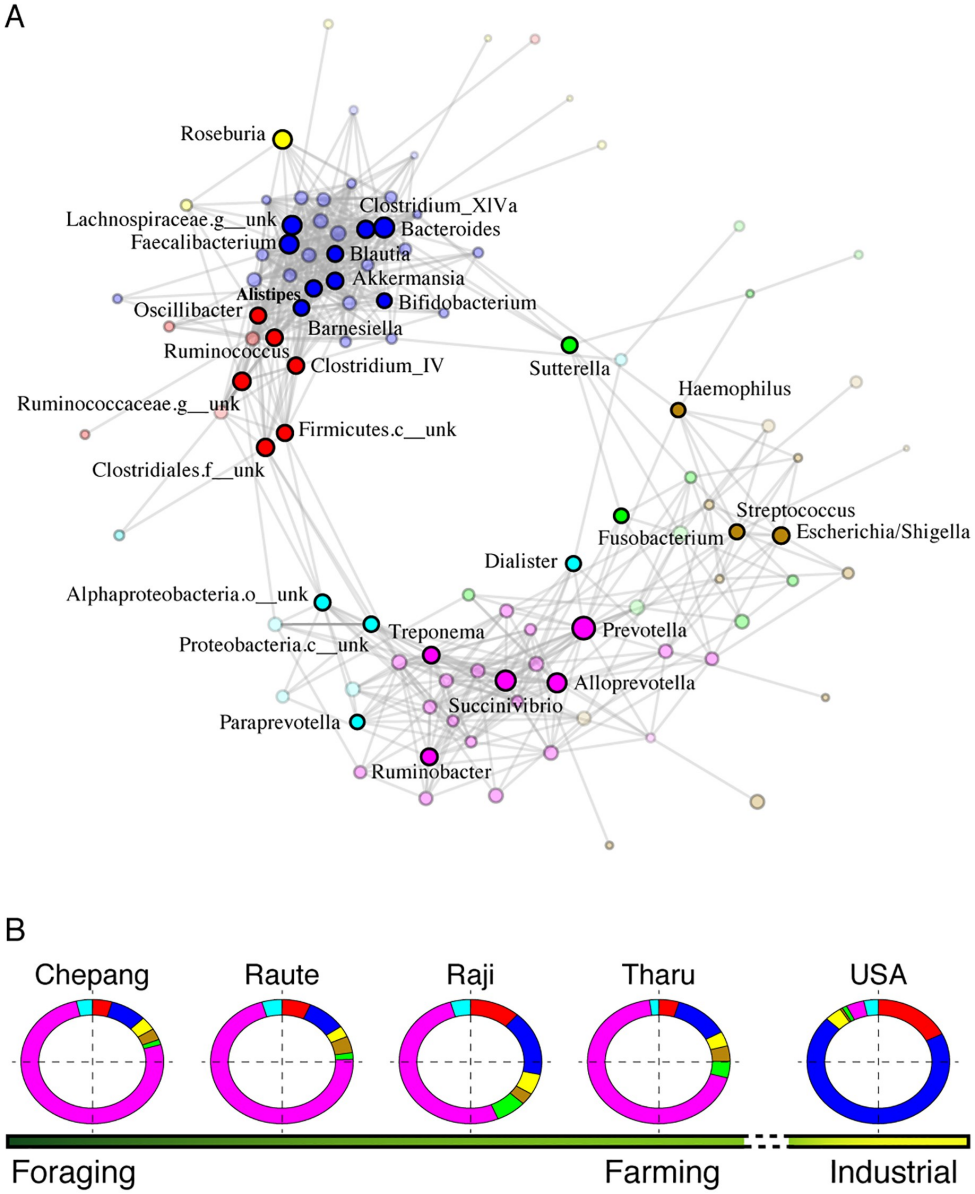
Microbial coabundance networks across lifestyles. (A) Visualization of the occurrence patterns of bacterial genera using the Fruchterman–Reingold force–directed layout algorithm. Nodes (circles) represent bacterial genera, node colors represent the seven CAGs, and node sizes represent genus abundance. Only the most dominant genera in each CAG are labeled. Edges represent the significant and positive correlations between genera. Members of the red, blue, and yellow CAGs are tightly correlated to one another and mostly negatively correlated with the members of cyan, magenta, gold, and green CAGs. Labels with “x__unk” indicate taxa with unknown classification level. (B) The relative proportions of these CAGs vary across the lifestyle gradient. Chepang foragers show elevated proportion of the magenta CAG, which is dominated by *Prevotella*, *Succinivibrio*, *Ruminobacter*, and *Treponema*. This CAG decreases in the Raute, Raji, and Tharu farmers with concurrent increase in the blue CAG, which is dominated by *Bacteroides*, *Faecalibacterium*, and *Bifidobacterium*. The American gut is dominated by the blue CAG and highly depleted of the magenta CAG. The data underlying this figure can be found in S1 Data. c, class; CAG, coabundance group; f, family; g, genus; o, order.

In addition to the individual taxa that differ across subsistence strategies, we wanted to determine whether microbial networks are also associated with lifestyle differences [24,43,44]. To understand how the gut microbiome network structure varies across these populations, we calculated the correlations between all pairs of bacterial genera in the gut using Sparse Correlations for Compositional data (SparCC) [45]. Clustering based on these correlations revealed seven bacterial coabundance groups (CAGs, S12 Fig). The dominant genera that defined these CAGs are *Ruminococcus*, *Bacteroides*, *Roseburia*, *Escheria/Shigella*, *Suturella*, *Prevotella*, and *Dialister* (Fig 6A and S13 Fig). These seven CAGs showed two antagonistic clusters: one cluster contains CAGs defined by *Ruminococcus*, *Bacteroides*, and *Roseburia*, and the second cluster contains CAGs defined by *Prevotella*, *Escheria/Shigella*, and *Dialister* (S13 Fig). Notably, the CAG dominated by *Prevotella* is most prominently represented in the Chepang and Raute, while members of the CAG dominated by *Bacteroides* are elevated in the Raji and Tharu (Fig 6B). Within the *Prevotella* CAG, *Treponema* and *Ruminobacter* are characteristic of the Chepang foragers. Conversely, the American gut is highly depleted of the *Prevotella* CAG and is dominated by the *Bacteroides* CAG. The results suggest how these changes in the microbiome that accompany lifestyle transitions may be viewed both at the level of individual taxa as well as higher-order community structure. Transitions from foraging to farming in Himalayan populations show changes in gut microbial networks, which appear to become more profound in industrialized societies.

### Factors associated with gut microbiome composition in the Himalaya

**Fig 7:**
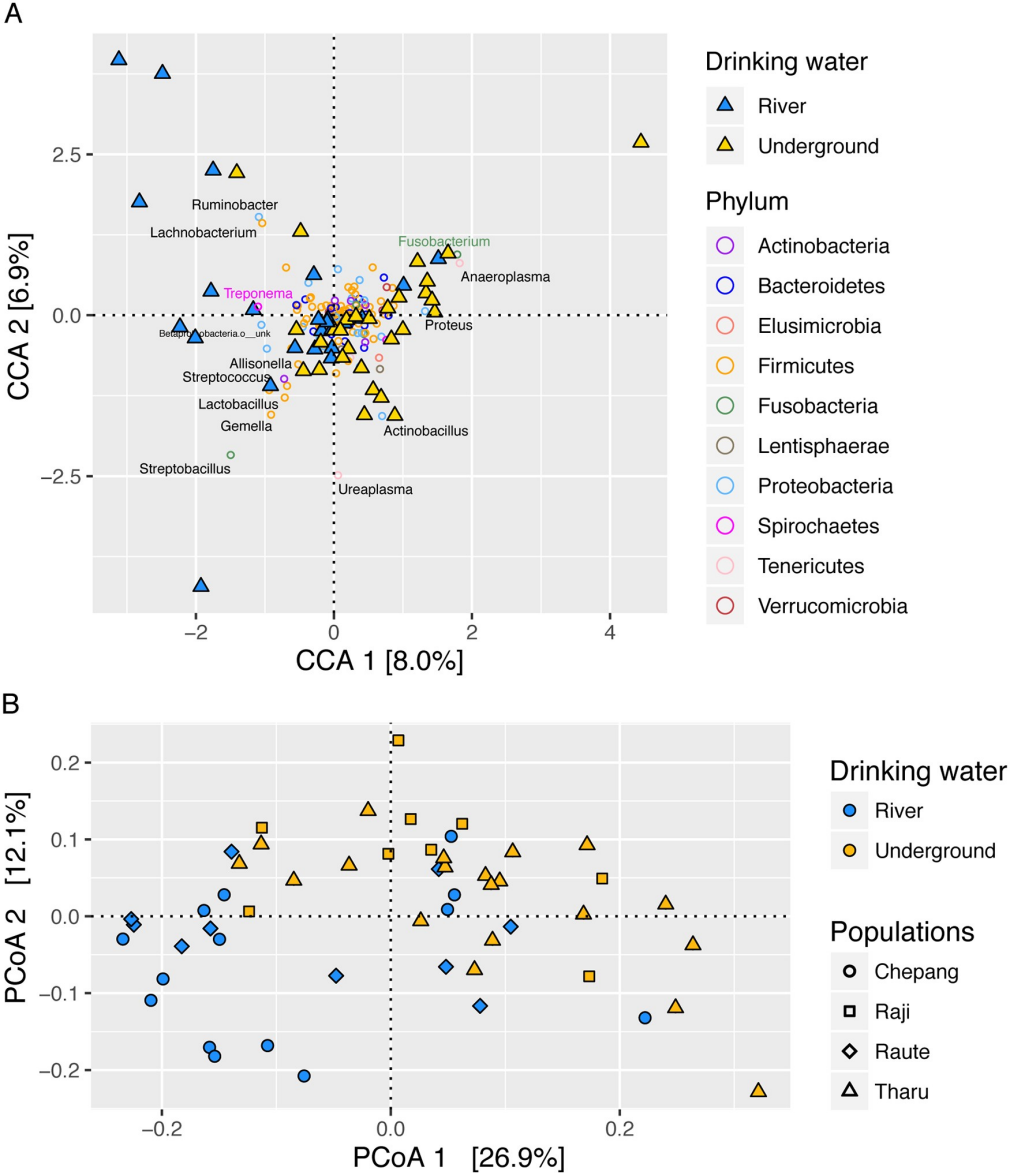
Gut microbiome composition is associated with environmental factors in Himalaya. (A) The two primary CCA axes and the proportion of constrained variance they explain are shown. Triangles represent individuals and circles represent genera. Individuals and genera are color coded by their respective drinking water sources and phyla. Drinking water and cooking fuel contributed most to CCA1, and *sisnu* (nettles) contributed most to CCA2. Genera labeled in grey contribute to the top two CCA axes. Among these, *Fusobacterium* and *Treponema* were significantly associated with drinking water. (B) PCoA of the unweighted UniFrac distances. Each dot represents an individual, colors indicate the two drinking water sources, and shapes represent different populations. Gut microbiomes of the Chepang (circles) and Raute (diamonds) who drink water from rivers and streams vary significantly from those of the Raji (squares) and Tharu (triangles). Statistical significance was assessed using PERMANOVA using the 10 variables that differentiate their lifestyles (*P* value = 0.0001). The data underlying this figure can be found in S1 Data and S4 Table. CCA, canonical correspondence analysis; PCoA, Principal Coordinates Analysis; PERMANOVA, permutational multivariate analysis of variance.

We next assessed whether any of the 10 dietary and environmental factors that differentiate the Himalayan populations (from Fig 2) correspond to the variation in gut microbiome composition. A canonical correspondence analysis (CCA) revealed that the 10 factors collectively explain 28% of the gut microbiome variation within Himalaya, while 72% of the variation remained unexplained. Of the 10 variables, the source of drinking water and use of solid biomass fuel were significantly associated with the gut microbiome composition in the Himalayan populations (*P* value = 0.009 and 0.028, respectively; permutation test). Both of these factors contributed most to the first CCA axis (CCA1), which distinguished the Chepang and Raute individuals who drink river water and exclusively burn solid biomass fuel for cooking from the Raji and Tharu who drink underground water and use biogas for cooking (Fig 7). As an alternative approach, we assessed associations between the gut microbiome composition of the Himalayan individuals (using Bray–Curtis, unweighted UniFrac, and weighted UniFrac) and the 10 lifestyle-associated factors by performing a PERMANOVA (S14 Fig). These analyses also revealed that drinking water was strongly associated with the gut microbiome variation within Himalaya (*P* = 0.001 for all three distances; effect sizes = 0.096, 0.1095, and 0.088 for Bray–Curtis; unweighted UniFrac; and weighted UniFrac distances, respectively). Individuals who drank river water had higher abundances of *Treponema*, and those who drank underground water had elevated levels of *Fusobacterium* (FDR adjusted *P* value = 0.01 and 0.003, respectively; Mann–Whitney test). Although cooking fuel was significantly associated with overall composition, none of the individual genera reached statistical significance after correcting for multiple testing.

To assess whether the association between gut microbiome and drinking water extend beyond the Nepali populations, we reanalyzed an independent 16S rRNA amplicon data set from Hadza hunter–gatherers from the Hukamako camp (*N* = 60) [21]. In the late dry season, the Hadza use water from two distinct sources—springs (*N* = 22) and streams (*N* = 38). We used a CCA to assess the associations between the gut microbiome of the Hadza and several dietary and environmental factors, including 72-hour recall of baobab, berries, honey, maize, meat, and tuber consumption; alcohol and cigarette use; as well as differences in drinking water sources (S8 Table). These variables collectively explained 16.5% of the gut microbiome variation in the Hadza gut microbiome. Among the variables used in the CCA, difference in drinking water source was most strongly associated with the Hadza gut microbiome composition followed by honey consumption (*P* = 0.0001 and 0.03, respectively; permutation test; S15 Fig). We also performed a PERMANOVA to assess associations between the gut microbiome composition of the Hadza individuals and their dietary and environmental factors using Bray–Curtis, unweighted UniFrac, and weighted UniFrac distances. All three analyses revealed the association between drinking water source and the Hadza gut microbiome (*P* = 0.001, 0.002, and 0.003, PERMANOVA; effect sizes = 0.051, 0.051, and 0.059 for Bray–Curtis, unweighted, and weighted UniFrac distances, respectively; S15 Fig). Therefore, our results in the Hadza and the Himalayan populations suggest that drinking water is strongly associated with the human gut microbiome and emphasize the need for additional work to elucidate the mechanisms by which drinking water may influence the gut microbiome.

## Discussion

Several previous reports show that gut microbiomes of traditional populations vary from those of populations living industrialized lifestyles [15,16,18–20,24–27,29,30]. These studies have emphasized that gut bacterial composition differs between these populations, alpha diversity is higher in traditional populations, and diet may be the primary driver of variation in the human gut microbiome. Contrary to previous studies, our work focuses on how the extent of departure from a foraging lifestyle may affect the human gut microbiome. In this study, we compared the gut microbiome from four rural Himalayan populations that led nomadic lifestyles until recently and transitioned to farming at various time points in the last 300 years. Although the individuals in our study have historically cohabited a geographically small region (less than 150,000 sq. km) in the Himalayan foothills and shared similar diets until recently, their current diets and lifestyles vary. Our results demonstrate that their gut microbiota strongly mirrors their lifestyles, indicating that the human gut microbiome can undergo pronounced changes within a short time (decades) of departure from foraging, as seen in the Raute and Raji. As dependences on agriculture increases, these changes become more pronounced, as seen in the Tharu. Since these populations cohabit comparable latitudinal regions, such changes in the gut microbiota are unlikely to be confounded by geography. Therefore, our findings suggest that a range of lifestyle changes more subtle than those associated with industrialization are strongly associated with alterations of the gut microbiome.

The gut microbiome variation between the Himalayan populations is consistent with the general patterns observed in many traditional human populations. More importantly, our results suggest certain genera represent conserved gut microbial markers of human subsistence states (S16 Fig). Previous studies of the industrialized gut community have demonstrated that microbiome composition associates with and can be driven by differences in host diet [4–6,8,15,17,21,27,46]. Several genera, including *Ruminobacter* and *Treponema*, that are associated with metabolizing uncultivated plant products and are enriched in the Chepang foragers in this study are also elevated in hunter–gatherers around the world [19,21,24,29]. Moreover, *Prevotella* and *Eubacterium*, which have been previously associated with vegetarian diet in the industrialized microbiome [5], were enriched in all Himalayan populations relative to Americans. In contrast, taxa associated with animal proteins in diet such as *Bacteroides* and *Blautia* [5,43] were enriched in the Americans relative to Himalayan populations. Notably, dietary animal protein content is low across Nepal [47].

In addition to diet, environmental factors may also influence the human gut microbiome [7,28,48]. Consistent with these findings, we found that differences in sources of drinking water are associated with gut microbiota composition in these Himalayan populations as well as Hadza hunter–gatherers, although additional work is needed to establish causality and to understand the mechanism by which drinking water may influence the gut microbiome. Drinking water contains a plethora of minerals and chemical compounds that influence human physiology [49]. Mineral and chemical contents in drinking water may differ by water source, which may alter the gut environment, thereby influencing the gut microbes. Moreover, the microbiome community in the drinking water may also vary between different sources. Recently, we have reported that different sources of Hadza drinking water contain a diverse set of bacterial taxa including families that are also found in the gut, such as the Prevotellaceae and the Spirochaetaceae [50]. Another recent study found surface water exposed to human and animal activities may contain higher levels of human commensal bacteria [22]. Microbes within drinking water may colonize the human gut or influence the resident microbial ecology during transit. Differences in mineral and microbial content in drinking water have also been previously reported in Nepal [51–54]. Furthermore, the chemical components in drinking water may interact with components of food [49], and the impact of such interactions on the complex gut ecosystem is currently unknown. Additionally, we found an association between gut microbiome composition in Himalayan populations and their use of solid biomass cooking fuel, which produces high levels of particulate matter. Prolonged inhalation of polluted air can influence the gut microbiome in mice [48]. In addition, intestinal parasite load has been shown to alter gut microbiota [28]. The association between gut microbiome and parasite load approached significance in our participants as well (*P* = 0.075, permutation test), although it did not reach significance likely due to lower parasite abundance in our participants.

Despite noticeable differences in the gut microbiome composition, we did not observe significant differences in gut bacterial diversity (alpha diversity) across lifestyles in the Himalayan populations. This finding is consistent with previous studies that compared populations that reside in similar geographical areas but practice different subsistence strategies such as the BaAka hunter–gatherers and Bantu farmers [19], Matses hunter–gatherers and Tunapuco farmers [29], as well as Bassa farmers and urban Nigerians [22]. These results collective indicate that difference in lifestyle alone is unlikely to generate differences in alpha diversity of the gut. However, these and other traditional populations such as the Hadza [24] have elevated gut bacterial diversity relative to the industrialized populations used as comparators in the respective studies. Some of these studies have also found lower interindividual variation within traditional societies compared to Western populations [22,29]. Neither the interindividual variation nor the alpha diversity differed between the Himalayan populations and Americans included in this study. The lack of differences in alpha diversity could be ascribable to geography as previously hypothesized [31]. Macroecological features, roughly corresponding latitude, may be an important factor that influences gut bacterial diversity in humans. The traditional populations included in previous studies reside in the tropical climate zones, which have higher macroecological biodiversity likely affecting both diet and environmental microbial exposures. In contrast, the Americans and Nepalis in this study reside in comparable nontropical latitudes (37.44 °N for Palo Alto and 26.97–29.15 °N for Nepal). Integration of data from multiple studies to examine alpha diversity trends is difficult due to technical differences in sample storage/preparation and batch effects of data generation. Future studies focused on people living in both temperate and tropical climates across a range of subsistence strategies are needed to provide further insight into how lifestyle and environment influence gut microbiota alpha diversity.

Our results provide key insights into how the extent of departure from a foraging lifestyle can impact the gut microbiome within the context of traditional, preindustrialization lifestyles but also reveal some limitations. Future important work includes determining how the transition from farming to industrialization may influence gut microbes. Ideally, such a study would compare the individuals living traditional lifestyles versus those from the same ethnic groups that have shifted to industrialized lifestyles, using metrics and surveys to quantify aspects of diet, lifestyle, and medical practices. Increasing sample sizes in future studies may provide more statistical power, although it can be difficult because most traditional populations across the world exist in small numbers. For example, in our study, the total population of Raute, including newborns and children, is 650. The Raji and Chepang exist as fragmented tribes in small and extremely remote villages within Himalaya that are separated by large geographical barriers. In this study, each village consists of a few hundred individuals from which we carefully sampled individuals with different grandparents, further reducing the number of participants. Even with small sample sizes, studies such as ours that focus on traditional populations have the potential to address key gaps in the field of human microbiome science.

In conclusion, our results emphasize the need to study additional traditional populations to understand how geography, climate, diet, and environment affect the gut microbiome. The global trends of bacterial taxa within the gut that undergo depletion or enrichment upon lifestyle transitions are striking. Incorporating metagenomics to characterize the gut microbial variation at finer scales, metabolomics and strain culturing to assess functional differences, and immune and metabolic profiling of these populations may reveal the functional consequence of these changes, both in terms of the intrinsic microbial ecology of the gut and the impact on human biology. Pursuit of mechanisms by which the gut microbiome interacts with human biology may reveal conserved connections with large implications for industrialized humans who lack these microbes that may have been part of our species’ evolution.

## Materials and methods

### Ethics statement

This work was approved by the Ethical Review Board of the Nepal Health Research Council (NHRC) as well as by the Stanford University Institutional Review Board.

### Study sites, participating individuals, and sample collection

Stool samples were collected with informed consent from 56 genetically unrelated adult participants (over 18 years old with different grandparents) from four indigenous Himalayan populations from Nepal and 10 adult Americans of European descent. Indigenous populations from Nepal included Chepang (*N* = 14), Raji (*N* = 10), Raute (*N* = 12), and Tharu (*N* = 20) inhabiting Chitwan, Bardia, Dadeldhura, and Sarlahi districts, respectively. The samples were collected in winter of 2016 (March and April) with consent from all participants.

In addition to collecting the fecal samples, we also obtained ethnolinguistic, demographic, environmental, and dietary data from the Himalayan participants using a survey questionnaire specifically designed for this study. The survey questionnaire assessed participants’ age, gender, diet, health status, use of medication, and behavioral practices such as tobacco and alcohol consumption, along with several environmental variables (S2 Table). In addition, we also visually inspected the stool samples of each individual under the microscope for the presence of intestinal parasites (triplicate slides per individual). Participants’ responses to survey data questionnaires are included in S4 Table.

### DNA extractions

Freshly produced stool samples from the Himalayan participants were collected on a clean OMNIgene gut accessory collection paper (OM-AC1). About 500 mg of the stool samples was transferred to the OMNIgene gut kit collection tube containing the stabilizing buffer using the clean spatula provided with the kit. The tubes were shaken hard in a back and forth motion until the fecal samples were completely homogenized. Tubes were transported at room temperature within 48–72 hours of collection to the Tribhuvan University Institute of Medicine, Kathmandu, Nepal, where they were transferred to −80 °C until DNA extraction. DNA was extracted using a MolBio Power Soil Kit according to the manufacturer’s protocol. Extracted DNA was shipped to Stanford University on dry ice and stored at −20 °C until sequencing. Samples from Americans were collected from volunteers at Stanford University in 15-ml centrifuge tubes and transported to the laboratory on ice. Half of each sample was immediately frozen at −80 °C. From the other half, 500 mg stool was transferred to OMNIgene collection tubes and kept at room temperature for 48–72 hours after which they were stored at −80 °C. DNA was extracted from both sets of samples simultaneously using the MolBio Power Soil Kit according to the manufacturer’s protocol and stored at −20 °C until sequencing.

### 16S rRNA gene amplicon sequencing and analyses

The V4 region of the 16S rRNA gene was PCR amplified using the primers and protocols described previously [55]. The amplified DNA fragments were multiplexed and subjected to paired-end sequencing using Illumina MiSeq. Of the 66 samples, one yielded very low levels of DNA and another failed the paired-end sequencing. After discarding these two samples, the final data set included 64 individuals (14 Chepang, 9 Raji, 11 Raute, 20 Tharu, and 10 Americans). The amplification primers and barcodes used for multiplexing are described in S4 Table.

Paired-end reads were processed using DADA2 [56] and subsequently analyzed in R using phyloseq [57]. In order to identify high quality sequences, reads were trimmed to 150 bp. Sequences with *N* nucleotides and/or >2 expected errors were discarded (max*N* = 0, maxEE = 2, truncQ = 2), and sequence variants were inferred by pooling reads from all samples (pool = TRUE). Sequence tables were then created by merging paired-end reads. A naïve Bayesian classifier method [58] implemented in DADA2 algorithm was used to assign taxonomy using the RDP v14 training set [59]. Multiple alignment was conducted using DECIPHER [60] package in R, and a maximum likelihood phylogenetic tree was constructed using phangorn [61], with a neighbor-joining tree as the starting point.

A total of 1,183,760 merged reads passed quality control, and 1,630 taxa were initially identified. After removing chimeric sequences, which constituted 22% of the reads, 921,345 merged reads remained. Further elimination of low-abundance phyla—Synergistetes and Deferribacteres—that were observed only once across all samples resulted in 883 taxa in the data set. After quality control, mean (±SD) sequencing depth per sample was 11,570 (±4,653). We performed three technical replicates of the frozen sample for one individual and a total of five replicates for two additional individuals for the OMNI samples. Since we did not observe marked differences in the technical replicates (S3 Fig), we retained the sample with highest coverage for these individuals. After removing the replicate samples, 64 individuals and 875 taxa remained in the final data set.

### Hadza sample collection and 16S sequencing

Stool samples and dietary recall from 60 Hadza individuals were conducted in the field at the time of sampling with the aid of an interpreter. Following informed consent, each participant provided a list of the plants, animals, and animals products consumed over the previous 72 hours, including alcohol and cigarette use. Location and type of water source was also recorded. Although the Hadza consume water primarily from a single source near their camp, foraging activities often take subjects several kilometers away from camp where their water source may vary. Raw 16S reads from the Hadza were previously published [21] and were processed using DADA2, as described above. This data set included 1,038,333 nonchimeric reads from 60 individuals that were assigned to 1,511 taxa.

### Random forest classifier model

A random forest classifier with 5,000 trees was constructed using all 35 variables (S3 Table) from the survey data. The R-package randomForest [62] was used to build the trees, and its “tuneRF” function was used to assess the optimal number of variables randomly sampled as candidates at each split. (“mtry” parameter, mtry = 6 for survey data). We also repeated these analyses on the 16S data using the RSVs as features and using mtry = 29 as determined by the tuneRF. The R-package “pROC” [63] was used to calculate and plot the area under the receiver operating characteristic curves (AUC) for each of the populations. In addition, brier skill scores (BSSs) in R-package “verification” [64] was used to assess the calibration of the RF model.

### Statistical analyses

Intestinal pathogens and all 35 variables recorded from the Himalayan populations were used to perform CA of the survey data using “FactoMineR” package in R [65]. Associations between rows and columns in the correspondence analysis were evaluated by performing a chi-squared test (*P* = 3.9 × 10^−6^). The contributions of each factors to the top two dimensions of CA were visualized using the “fviz_contrib” function in R-package factoextra [66]. The expected contribution to the top two dimensions under a uniform model was determined, and factors that contributed more than the expected were considered important in differentiating lifestyles.

CCA was performed in the Himalayan populations using the 10 variables that differentiated lifestyles in the correspondence analysis by calling the “cca” function from vegan package [67] via phyloseq. For the Hadza, CCA was performed using nine variables including six dietary variables (baobab, berries, maize, tubers, honey, and meat consumption in the last 72 hours), alcohol and cigarette consumption, as well as source of drinking water. RSV counts were used as features of gut microbiomes in both the Himalayan and the Hadza populations. Permutation tests with 10,000 permutations were performed to evaluate the significance of each CCA model and terms using “anova.cca” function in “vegan.” For all CCA models, the *P* values from the permutation tests were less than 0.05, indicating that the CCA model explained more variance of the gut microbiome in the Himalayan and the Hadza populations than expected by chance. American samples were excluded for both CA and CCA analyses.

Phylogenetic diversity was computed by rarefying the samples to various depths starting from 10–6,500 sequences per sample. Alpha diversity was measured using species richness, Shannon’s H, Simpson’s D, and Fisher’s alpha calculated as the mean values from 100 iterations at each depth. Kruskal–Wallis tests were used to assess the significance of differences in each of the alpha diversity metrics between populations at each rarefaction depth. Dunn’s posthoc test was performed to assess pairwise differences between populations. Differences in rarefaction depth did not alter significance of the observed differences. A generalized linear mixed effect model was used to evaluate associations between the 10 dietary and environmental factors and the two metrics of alpha diversity. Four models were created, each with the four metrics of alpha diversity (observed species, Shannon’s H, Simpson’s D, and Fisher’s alpha) as the response variables; the 10 factors were treated as explanatory variables with fixed effects; and each individual had random effect. Beta diversity was assessed using Bray–Curtis as well as unweighted and weighted UniFrac distances calculated by log transformation of the nonrarefied 16S count data. PERMANOVA was performed using the vegan package in R [68]. For all PERMANOVA analyses, 10,000 randomizations were performed to assess the statistical significance. In order to identify differentially abundant taxa at the phylum and genus levels, we first agglomerated the taxa abundance (counts) at each taxonomic level, respectively. The differences in taxa abundance (counts) were then assessed using the DESeq2 package [42].

SparCC was used to assess correlations between bacterial genera, as described previously [45]. SparCC is specifically designed to measure correlations in microbiome data and computes compositionality robust correlations by averaging multiple iterations of data. The statistical significance of the inferred correlations is then assessed using a bootstrap procedure. First, a large number of simulated data sets, in which all components are uncorrelated, are generated. Then, correlations are inferred from each simulated data set with the same parameter setting as is used for the original data. Finally, for each component pair, pseudo *P* values are assigned to be a proportion of simulated data sets for which a correlation value is at least as extreme as the one computed for the original data. We computed bacterial correlations for all pairs of genera after removing genera with less than 2 reads in at least 5% of samples (3 individuals and 124 genera). Correlations were computed from 100 iterations of the data, and we repeated the iterative procedure 100 times to compute the *P* values. *P* values < 0.05 after multiple testing correction were considered significant. Bacterial networks were visualized using the Fruchterman–Reingold force–directed layout algorithm implemented in the igraph package in R [69].

All multiple testing corrections were performed by computing FDRs using the Benjamini–Hochenberg method, and adjusted *P* values < 0.05 were considered statistically significant. A phyloseq object containing the 16S data and metadata as well as the analyses protocols used in this work are included in the supplementary data.

## Supporting information

S1 Fig Evaluation of the RF classification model of populations based on lifestyles. AUCs were computed to evaluate the performance of the RF model for each population. The AUC values for all four populations are close to 1.0, indicating that the RF model was able to accurately distinguish individuals based on their lifestyles. As a second metric, the BSS evaluates the calibration of the RF models, i.e., whether the predictions made by the RF are more reliable than randomly assigning the individuals to a particular population. A BSS of 1 would indicate perfect calibration. A BSS of 0 means no improvement compared to random assignment, while a negative BSS would suggest worse than random assignment. A BSS > 0.5 for all four populations suggests high accuracy of the RF model. AUC, area under the receiver operating characteristic curve; BSS, brier skill score; RF, random forest.
(TIF)

S2 Fig Dietary and environmental factors associated with lifestyle gradients in the Himalaya. Several dietary factors distinguished the four Himalayan populations included in this study. Foraged plants such as *sisnu* (nettles) and *jaand*, a slushy alcoholic beverage made from fermenting millet or corn, are staples of the Chepang diet. Although not recorded in the survey data, our Chepang participants reported that due to lack of irrigation, they are unable to grow rice and are limited to growing crops that require less water such as buckwheat, millet, and corn and forage for tubers (*gittha vyakur*) in the forest. In contrast, alcohol use was minimal among the Raute, Raji, and Tharu. Moreover, perceived food scarcity was higher in the Chepang and Raute, both of which reside in remote villages relative to the Raji and Tharu. Although meat consumption was low across all four populations, the Tharu consumed animal products such as yogurt most frequently. According to our Tharu participants, *ghonghi* (snails) are staples in their diet, although this dietary parameter was not included in our survey. In addition to diet, several environmental factors also differed across the Himalayan populations. The Chepang and Raute who reside in remote villages still fetch their drinking water from rivers and streams. Conversely, Raji and Tharu who reside in more urbanized areas have installed tube wells in their homes, enabling access to underground water for drinking. The use of SBM was lower in the Tharu and Raji, as they frequently used NSBM such as biogas. Conversely, the Chepang and Raute are still completely dependent on burning firewood for cooking. Although we detect low overall levels of intestinal parasites in our participants, *Ascaris*, *Entamoeba*, *Trichuris*, *Hymenolepis*, and *Coccidia* appear in some individuals. Parasite infection was highest in the Chepang, intermediate in the Raute and Raji, and lowest in the Tharu. Smoking and tobacco consumption was higher in the Tharu and Chepang relative to Raji and Raute. NSBM, nonsolid biomass fuel; SBM, solid biomass fuel.
(TIF)

S3 Fig 16S sequencing and quality filtering. (A) Sequencing depth for each taxon and each sample before filtering. Over 1,600 RSVs were initially identified, but many were chimeric and detected by a single read. (B) Removal of chimera did not reduce sequencing depth for the taxa or for the samples. (C) Abundance of the phyla in the data set. Deferribacteres and Synergistes were detected in only a few individuals and were lowly abundant (read count < 4) and were removed. (D) After quality filtering of chimera and low-abundance taxa, 12 phyla and a total of 883 taxa remained in the data set. RSV, read sequence variant.
(TIF)

S4 Fig Comparison of frozen and OMNIgene samples. Since flash freezing of the samples was not possible in the remote sampling areas, we used commercially available DNAgenotek OMNIgene kits to collect stool samples from the four Himalayan populations. We also collected stool samples from 10 Americans of European descent from Palo Alto. We divided these samples into two sets, the first set was transferred into OMNIgene kits, and the second set was frozen at −80 °C. The OMNIgene kits containing the stool samples were kept at room temperature for 24–72 hours, then they were frozen at −80 °C. DNA extraction, 16S amplification (V4), and sequencing was performed simultaneously for both sets of samples. This allowed us to determine whether the kit collections in the field could faithfully reproduce microbiome profiles observed in freshly frozen stool. (A) Analysis of gut bacterial community using PCoA of unweighted and weighted UniFrac distances showed no significant differences between the sampling methods (*P* > 0.05 for both distances, PERMANOVA). Replicate samples from the same individual also tended to be in close proximity to one another in both analyses. (B) Alpha diversity assessed using species richness, Fisher index, and Shannon index was not significantly different between the two methods (*P* > 0.05, Kruskal–Wallis test). (C) Although comparison of frozen and OMNI samples showed few differences, abundance of Euryarcheota and Cyanobacteria/chloroplast were lower and higher in OMNI samples, respectively. Both constituted negligible fractions of gut bacteria and were removed from further analyses. (D) Comparison of differences in taxa abundances at the genus level using a negative binomial GLM for differential abundance analysis as implemented in DESeq2 demonstrated that none of the genera differed significantly between the sampling methods (FDR adjusted *P* values > 0.05). Hence, these results collectively demonstrate that sampling using OMNIgene kits did not introduce major biases in our data. DESeq2, Differential Expression analysis for Sequence count data version 2; FDR, false discovery rate; GLM, generalized linear model; PCoA, Principal Coordinate Analysis; PERMANOVA, permutational multivariate analysis of variance.
(TIF)

S5 Fig Differences in gut microbiome compositions across lifestyles. (A) Visualization using a PCoA of the Bray–Curtis (left) and weighted UniFrac distances (right). Each dot represents an individual, and colors indicate the populations. (B) Distribution of populations along the PCoA1 axis shows patterns of separation by lifestyles. Chepang foragers (red), Raute (yellow) and Raji (cyan), Tharu farmers (blue), and Americans (orange). (C) In both cases, PCoA1 was strongly correlated with CA1 obtained from the analysis of the survey data. Spearman’s Rho for Bray–Curtis and weighted UniFrac were 0.47 and 0.28, respectively (*P* < 0.05 for both, correlation test). Correlations between CA2 and PCoA1 were insignificant (*P* > 0.05 for both distances, correlation test). CA, correspondence analysis; PCoA, Principal Coordinate Analysis.
(TIF)

S6 Fig Visualization of distinctions in gut microbial communities across population using PCoA. PCoA of the unweighted and weighted UniFrac distances (top and middle, respectively) and Bray–Curtis distance (bottom). All four plots on each row differ only in coloring of the dots to help visualize the distribution of individuals in each population. PCoA, Principal Coordinate Analysis.
(TIF)

S7 Fig Variation in gut microbiota within Himalaya. Columns show PCoA of the three distance matrices of the four Himalayan populations after removing Americans from the analysis. Top row shows the top two PCoA axes and variance explained. Significant differences in gut microbiome composition within Himalaya were observed for all three distances (*P* < 0.05, PERMANOVA). Bottom row shows the distribution of the Himalayan populations along the PCoA1 axis. The separation between Chepang foragers (red) and Tharu farmers (blue) is the strongest within Himalaya; the two transitioning Raute and Raji populations are intermediates. PCoA, Principal Coordinate Analysis; PERMANOVA, permutational multivariate analysis of variance.
(TIF)

S8 Fig Evaluation of the RF classification model of populations based on 16S reads. AUCs were computed to evaluate the performance of the RF model for each population. AUC for all populations was calculated by averaging the AUC for each population. A second metric, the BSS, was used to assess calibration of the RF model. A BSS = 0 means RF model performance is no different than random assignment, a negative BSS would suggest worse performance than random assignment, and BSS > 0 indicates that the RF model performed better than a random assignment. A BSS score of 1 would indicate a perfect calibration. The AUC and BSS values for the Chepang, the Americans, and the Tharu are >0.90 and >0.3, indicating that the RF model was able to accurately differentiate individuals from these populations based on their gut microbiome. Low BSS scores for Raute and Raji suggest that the RF model was unable to accurately distinguish individuals from these populations based on their gut microbiome, consistent with two similar populations in a transition state. AUC, area under the receiver operating characteristic curve; BSS, brier skill score; RF, random forest.
(TIF)

S9 Fig Alpha diversity within Himalayan and American gut microbiomes measured using additional metrics. Rarefaction curves showing two additional commonly used measures of alpha diversity—Fisher’s alpha (top) and Simpson’s index (bottom) at different rarefaction depths (x-axes). No significant difference in Fisher’s alpha was detected across the five study populations. Simpson’s index was lower in the Tharu relative to the Americans, but none of the alpha diversity metrics showed significant differences between any of the four Himalayan populations.
(TIF)

S10 Fig Abundances of differentially abundant genera across populations. Each subplot shows abundance of an individual taxa in the five populations. Differentially abundant genera from Bacteroidetes (A), Proteobacteria (B), Verrucomicrobia (C), Spirocheates (D), Actinobacteria (E), Elusimicrobia (F), and Tenericutes (G). Labels with “c__unk” and “f__unk” indicate taxa with unknown class and family, respectively.
(TIF)

S11 Fig Complex patterns of differential abundances of Firmicutes across populations. Several genera are significantly enriched in the rural Himalayan populations, and others are depleted. Labels with “g__unk” and “o__unk” indicate taxa with unknown genus and order, respectively.
(TIF)

S12 Fig Heatmap showing clustering of genera based on coabundance patterns. Compositionality robust correlations between bacterial genera across all samples (*N* = 64) were calculated using SparCC. Genera with less than 2 reads in at least 5% of samples (three individuals) were removed from this analysis. Ward’s hierarchical clustering performed using the correlation metric distance revealed bacterial CAGs. Dendrograms show that bacterial genera cluster into seven CAGs. Genera labels are colored based on their CAG memberships. Labels with “x__unk” indicate taxa with unknown classification level. c, class; CAG, coabundance group; f, family; g, genus; o, order; SparCC, Sparse Correlations for Compositional data.
(TIF)

S13 Fig Bacterial coabundance networks. Significant associations between bacterial genera after adjusting for multiple testing (FDR adjusted *P* < 0.05) visualized using the Fruchterman–Reingold force–directed layout algorithm. Nodes (circles) represent bacterial genera, node colors represent the seven CAGs, and node sizes represent genus abundance. Edges represent the significant correlations between genera. (A) Edges colored in green and red show positive and negative correlations between the genera, respectively. Members of the red, blue, and yellow CAGs are mostly negatively correlated with the members of cyan, magenta, gold, and green CAGs. (B) Only the significant and positive correlations between bacterial genera are shown. Labels with “x__unk” indicate taxa with unknown classification level. c, class; CAG, coabundance group; f, family; FDR, false discovery rate; g, genus; o, order.
(TIF)

S14 Fig Gut microbiome associations with variations in drinking water sources in Himalaya. PCoA of the Bray–Curtis and weighted UniFrac distances. Each dot represents an individual, colors indicate the two drinking water sources, and shapes represent different populations. Gut microbiomes of the Chepang (circles) and Raute (diamonds) who drink water from rivers and streams vary significantly from those of the Raji (squares) and Tharu (triangles). Statistical significance was assessed using PERMANOVA using the 10 variables that differentiate their lifestyles (*P* value = 0.0001 for both distances). PCoA, Principal Coordinate Analysis; PERMANOVA, permutational multivariate analysis of variance.
(TIF)

S15 Fig Gut microbiome is associated with variations in drinking water sources in Hadza. (A) CCA biplot showing separation between gut microbiome of Hadza from the Hukamako camp; all samples from late dry season. Individuals who use spring and stream water are shown in yellow and green squares, respectively. Circles represent bacterial RSVs, and the proportions of constrained variance explained by the two primary CCA axes are shown. (B) The two primary principal coordinate axes of Bray–Curtis, unweighted UniFrac, and weighted UniFrac distances in the Hukamako Hadza late dry season samples are shown along with the fraction of variance they explain. Each dot represents an individual, and colors indicate the two drinking water sources. Statistical significance was assessed using PERMANOVA (*P* value < 0.05 for all three distances). CCA; PERMANOVA, permutational multivariate analysis of variance; RSV, read sequence variant.
(TIF)

S16 Fig Proposed dynamics of gut microbiome in lifestyle transitions. We propose that fluctuations in individual gut taxa show complex patterns as humans transition from one lifestyle to another. A few examples of bacterial taxa and their consistent patterns of changes in human populations around the world are shown. Certain genera such as *Treponema* and *Ruminobacter* that are characteristic of hunter–gatherers decline in agrarians and industrialists. In contrast, taxa such as *Alistipes* and *Akkermansia* increase in nonforagers. Genera such as *Bacteroides* show a gradual increase from foragers to industrialists and *Bulleidia* shows an opposite trend. Higher abundances of taxa such as *Prevotella* and *Succinivibrio* are characteristics of traditional lifestyles and are absent or rare in industrialists. Both dietary and environmental factors are likely to influence the gut microbiome. In this study, source of drinking water was strongly associated with gut microbiome composition. Other environmental factors such as parasite load and antibiotic usage also influence the gut microbiota.
(TIF)

S1 Table Populations, their subsistence strategies, and sample sizes. For the 10 Americans we compared frozen samples to those collected using OMNIgene collection kits. We also performed three technical replicate sequencing for two Americans.
(XLSX)

S2 Table Survey questionnaire. Survey data were collected for 53 of the 54 individuals from the four Himalayan populations. One individual consented to donating samples but was not interested in participating in the survey. We included the sample and removed this individual from the survey data analyses. Prolonged exposure to pollutants generated during combustion of solid biomass fuel such as firewood or animal dungs due to indoor cooking has the potential to alter gut microbiome. Hence, we assessed the fuel types used for cooking and location of kitchen in our Himalayan participants. We also inquired about the sources of drinking water among our participants. None of the participants filtered or purified water before drinking. Thus, this variable was excluded from analysis. We surveyed three replicates from each the stool samples under a microscope to identify parasites *Ascaris*, *Entamoeba*, *Trichuris*, *Hymenolepis*, and *Coccidia*. If any of these parasites were present, the individuals were labeled positive. Frequency of plant and animal products in diet were also recorded. Binary responses were coded as 0 and 3 and frequency variables were coded as 0, 1, 2, or 3 for least frequent to most frequent.
(XLSX)

S3 Table Random forest classifications. Summary of random forest classification using survey data (A) and 16S ASV table (B). Lowest out of bag error (3%) for the survey data was obtained with 2,750 trees, and lowest out of bag error (32%) for the 16S data was obtained with 1,950 trees. ASV, amplicon sequence variant.
(XLSX)

S4 Table Primers, sequencing depth, and survey data. Amplification primers, barcodes used for multiplexing, and sequencing depth for samples in this study, along with survey data collected from participants. “NA” indicates missing data.
(XLSX)

S5 Table Mean distances within and between populations. Average pairwise distances between individuals within and between populations computed using Bray–Curtis, unweighted UniFrac, and weighted UniFrac matrices.
(XLSX)

S6 Table Significantly different phyla across populations. Summary table of differential abundance of phyla (taxa collapsed based on phylum names) across the five populations. Statistical significance was assessed using a negative binomial GLM as implemented in DESeq2. Multiple testing corrections were performed by computing FDRs using Benjamini–Hochenberg method and multiple testing adjusted *P* values < 0.05 were considered statistically significant. DESeq2, Differential Expression analysis for Sequence count data version 2; FDR, false discovery rate; GLM, generalized linear model.
(XLSX)

S7 Table Significantly different genera across populations. Summary table of differential abundance of genera (taxa collapsed based on genus names) across the five populations. Statistical significance was assessed using a negative binomial GLM as implemented in DESeq2. Multiple testing corrections were performed by computing FDRs using Benjamini–Hochenberg method and multiple testing adjusted *P* values < 0.05 were considered statistically significant. DESeq2, Differential Expression analysis for Sequence count data version 2; FDR, false discovery rate; GLM, generalized linear model.
(XLSX)

S8 Table Hadza dietary and environmental data. Dietary and environmental factors of the Hadza individuals used in this study. Dietary data are based on 72-hour recall and include consumption of baobab, berries, honey, maize, meat, and tubers, as well as alcohol and cigarette use. Environmental factors include differences in drinking water sources.
(CSV)

S1 Data Phyloseq object. A phyloseq object containing ASV table, sample data, taxonomy table, and phylogenetic tree used in this study. ASV, amplicon sequence variant.
(RDS)

S1 Alternative Language Abstract A summary of the findings from this study in Nepali language as translated by Aashish R. Jha. (PDF)

## Abbreviations

ASV: amplicon sequence variant
BSS: brier skill score
CA: correspondence analysis
CAG: coabundance group
CCA: canonical correspondence analysis
DESeq2: differential expression analysis for sequence count data version 2
FDR: false discovery rate
GLM: generalized linear model
NHRC: Nepal Health Research Council
OM-AC1: OMNIgene gut accessory collection paper
OTU: operational taxonomic unit
PCoA: Principal Coordinates Analysis
PERMANOVA: permutational multivariate analysis of variance
rRNA: ribosomal RNA
SparCC: Sparse Correlations for Compositional data
