# Supplementary figures and images for: Gut microbiome transition across a lifestyle gradient in Himalaya
Source: PLoS Biol. 2018 Nov 15;16(11):e2005396. doi: 10.1371/journal.pbio.2005396 (PMC6237292; doi:10.1371/journal.pbio.2005396)

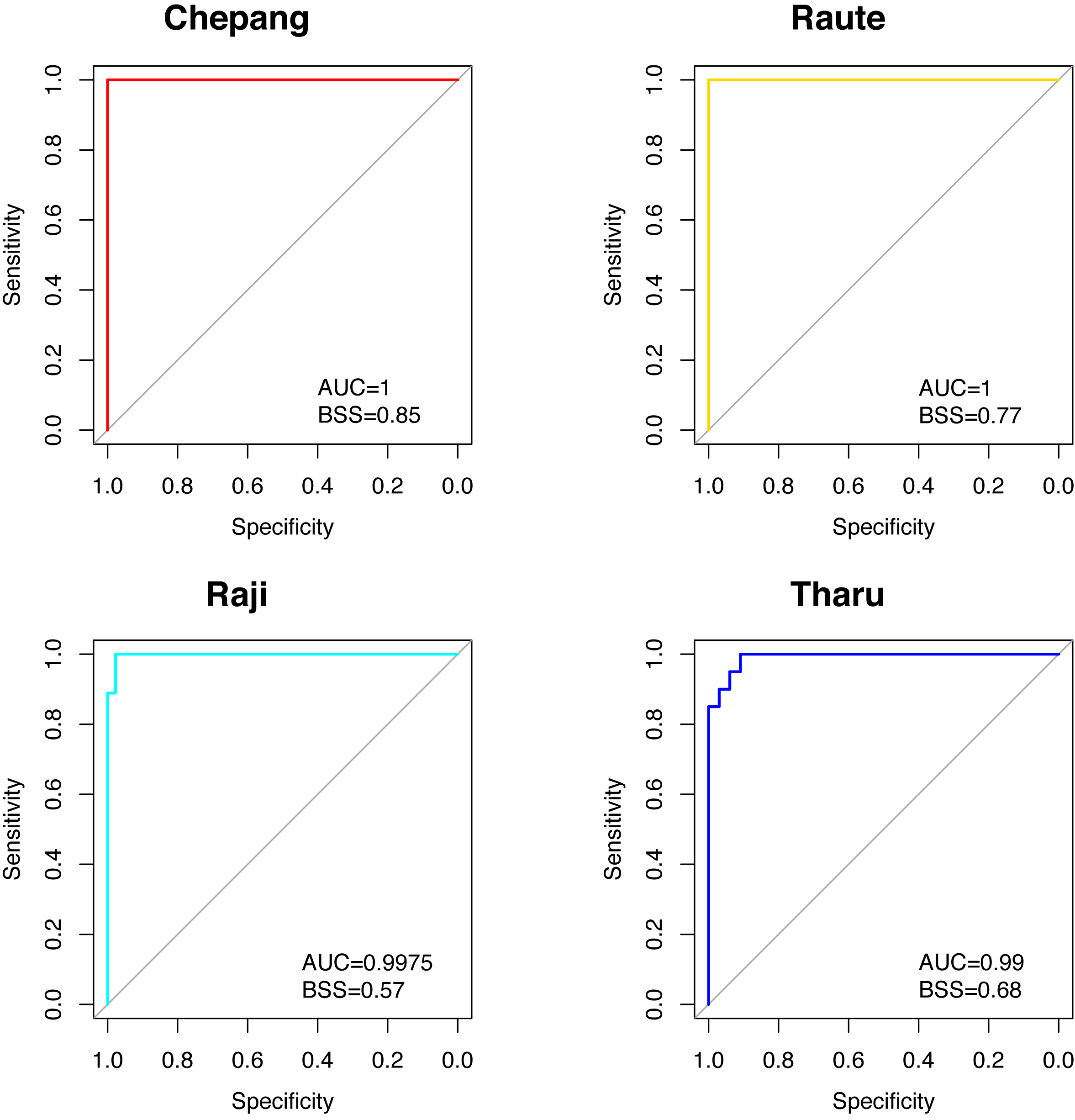

Supplement: S1 Fig — AUCs were computed to evaluate the performance of the RF model for each population. The AUC values for all four populations are close to 1.0, indicating that the RF model was able to accurately distinguish individuals based on their lifestyles. As a second metric, the BSS evaluates the calibration of the RF models, i.e., whether the predictions made by the RF are more reliable than randomly assigning the individuals to a particular population. A BSS of 1 would indicate perfect calibration. A BSS of 0 means no improvement compared to random assignment, while a negative BSS would suggest worse than random assignment. A BSS > 0.5 for all four populations suggests high accuracy of the RF model. AUC, area under the receiver operating characteristic curve; BSS, brier skill score; RF, random forest. (TIF) [file pbio.2005396.s001.tif]

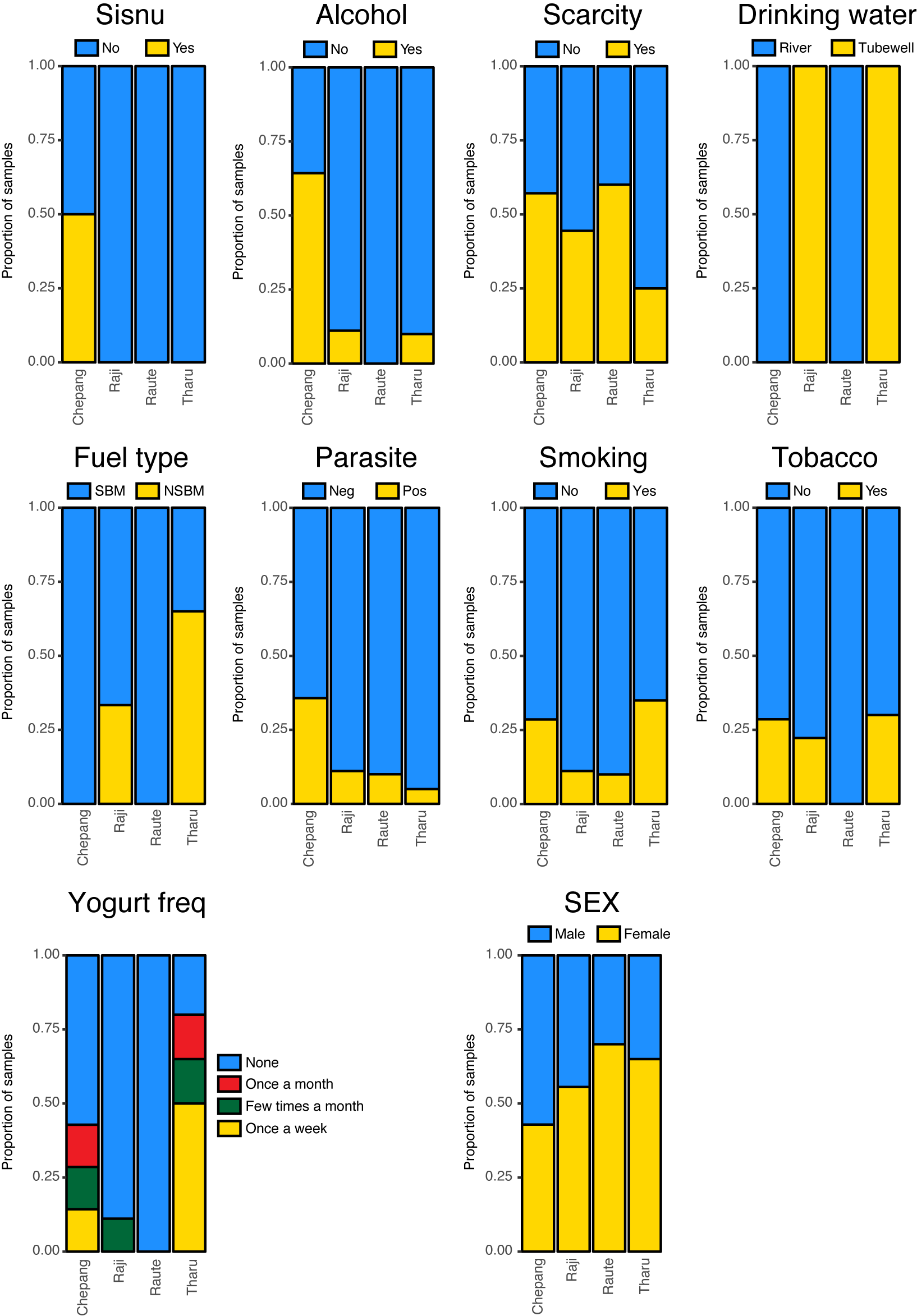

Supplement: S2 Fig — Several dietary factors distinguished the four Himalayan populations included in this study. Foraged plants such as sisnu (nettles) and jaand, a slushy alcoholic beverage made from fermenting millet or corn, are staples of the Chepang diet. Although not recorded in the survey data, our Chepang participants reported that due to lack of irrigation, they are unable to grow rice and are limited to growing crops that require less water such as buckwheat, millet, and corn and forage for tubers (gittha vyakur) in the forest. In contrast, alcohol use was minimal among the Raute, Raji, and Tharu. Moreover, perceived food scarcity was higher in the Chepang and Raute, both of which reside in remote villages relative to the Raji and Tharu. Although meat consumption was low across all four populations, the Tharu consumed animal products such as yogurt most frequently. According to our Tharu participants, ghonghi (snails) are staples in their diet, although this dietary parameter was not included in our survey. In addition to diet, several environmental factors also differed across the Himalayan populations. The Chepang and Raute who reside in remote villages still fetch their drinking water from rivers and streams. Conversely, Raji and Tharu who reside in more urbanized areas have installed tube wells in their homes, enabling access to underground water for drinking. The use of SBM was lower in the Tharu and Raji, as they frequently used NSBM such as biogas. Conversely, the Chepang and Raute are still completely dependent on burning firewood for cooking. Although we detect low overall levels of intestinal parasites in our participants, Ascaris, Entamoeba, Trichuris, Hymenolepis, and Coccidia appear in some individuals. Parasite infection was highest in the Chepang, intermediate in the Raute and Raji, and lowest in the Tharu. Smoking and tobacco consumption was higher in the Tharu and Chepang relative to Raji and Raute. NSBM, nonsolid biomass fuel; SBM, solid biomass fuel. (TIF) [file pbio.2005396.s002.tif]

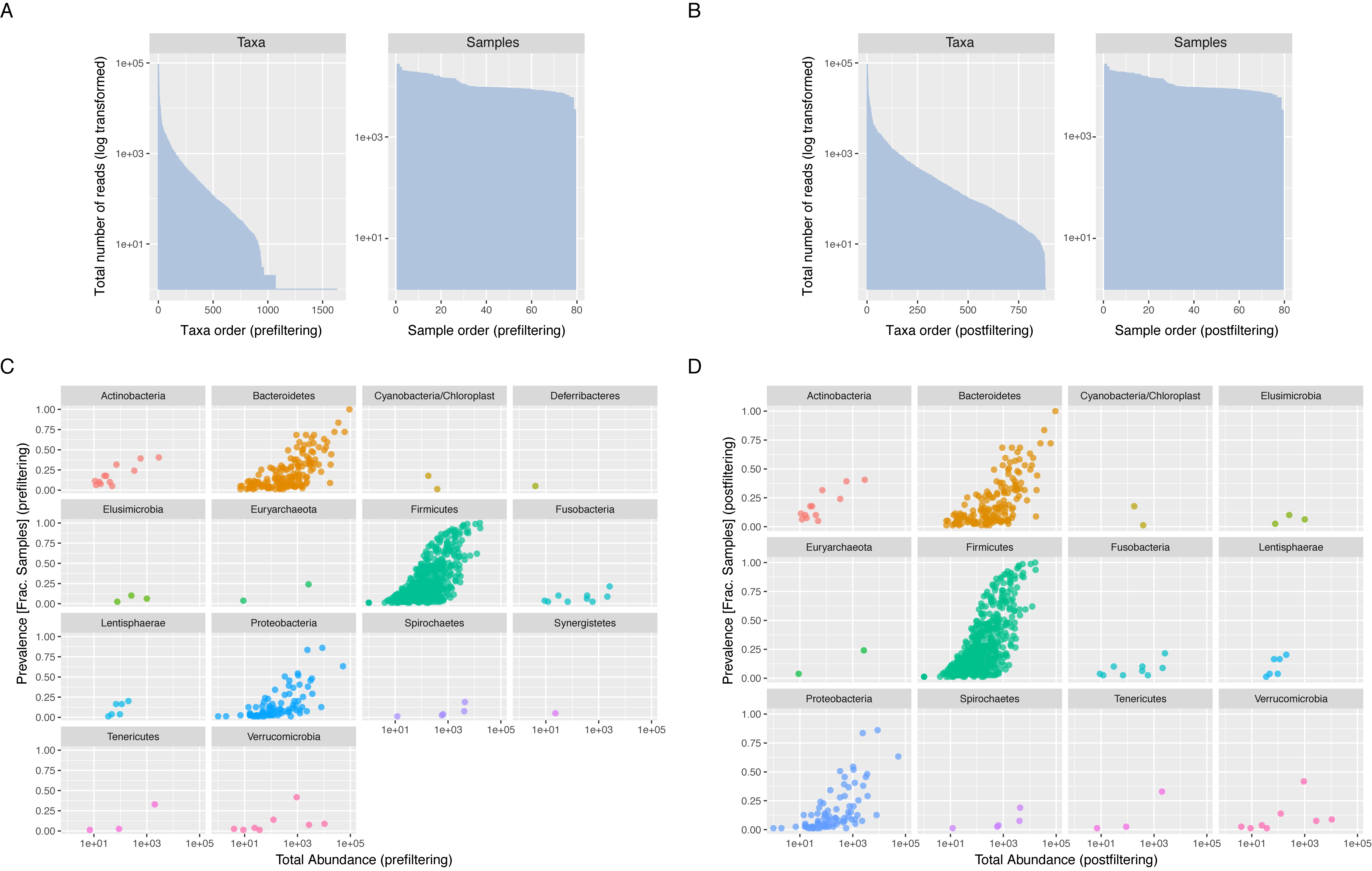

Supplement: S3 Fig — (A) Sequencing depth for each taxon and each sample before filtering. Over 1,600 RSVs were initially identified, but many were chimeric and detected by a single read. (B) Removal of chimera did not reduce sequencing depth for the taxa or for the samples. (C) Abundance of the phyla in the data set. Deferribacteres and Synergistes were detected in only a few individuals and were lowly abundant (read count < 4) and were removed. (D) After quality filtering of chimera and low-abundance taxa, 12 phyla and a total of 883 taxa remained in the data set. RSV, read sequence variant. (TIF) [file pbio.2005396.s003.tif]

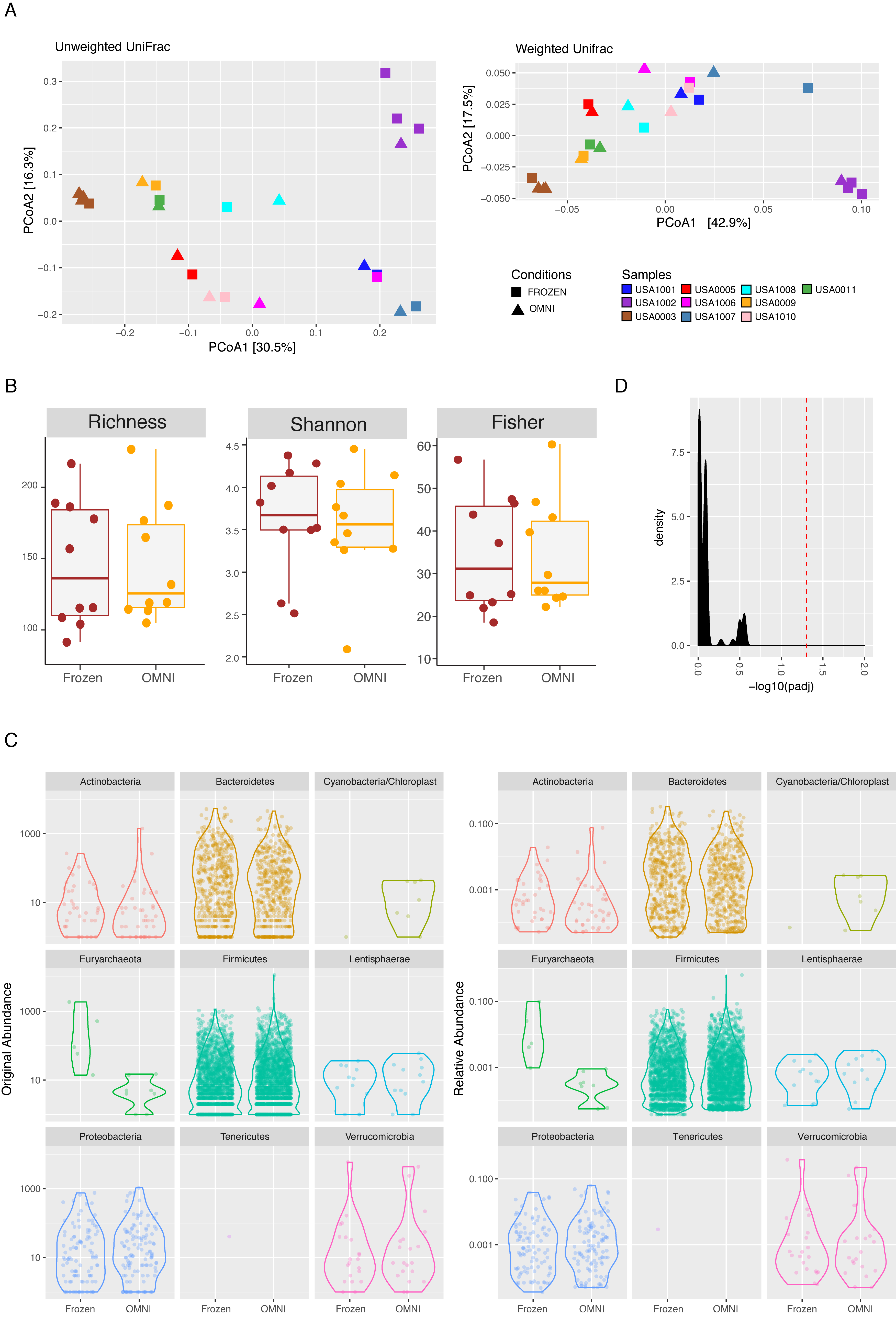

Supplement: S4 Fig — Since flash freezing of the samples was not possible in the remote sampling areas, we used commercially available DNAgenotek OMNIgene kits to collect stool samples from the four Himalayan populations. We also collected stool samples from 10 Americans of European descent from Palo Alto. We divided these samples into two sets, the first set was transferred into OMNIgene kits, and the second set was frozen at −80 °C. The OMNIgene kits containing the stool samples were kept at room temperature for 24–72 hours, then they were frozen at −80 °C. DNA extraction, 16S amplification (V4), and sequencing was performed simultaneously for both sets of samples. This allowed us to determine whether the kit collections in the field could faithfully reproduce microbiome profiles observed in freshly frozen stool. (A) Analysis of gut bacterial community using PCoA of unweighted and weighted UniFrac distances showed no significant differences between the sampling methods (P > 0.05 for both distances, PERMANOVA). Replicate samples from the same individual also tended to be in close proximity to one another in both analyses. (B) Alpha diversity assessed using species richness, Fisher index, and Shannon index was not significantly different between the two methods (P > 0.05, Kruskal–Wallis test). (C) Although comparison of frozen and OMNI samples showed few differences, abundance of Euryarcheota and Cyanobacteria/chloroplast were lower and higher in OMNI samples, respectively. Both constituted negligible fractions of gut bacteria and were removed from further analyses. (D) Comparison of differences in taxa abundances at the genus level using a negative binomial GLM for differential abundance analysis as implemented in DESeq2 demonstrated that none of the genera differed significantly between the sampling methods (FDR adjusted P values > 0.05). Hence, these results collectively demonstrate that sampling using OMNIgene kits did not introduce major biases in our data. DESeq2, Differential Exp [file pbio.2005396.s004.tif]

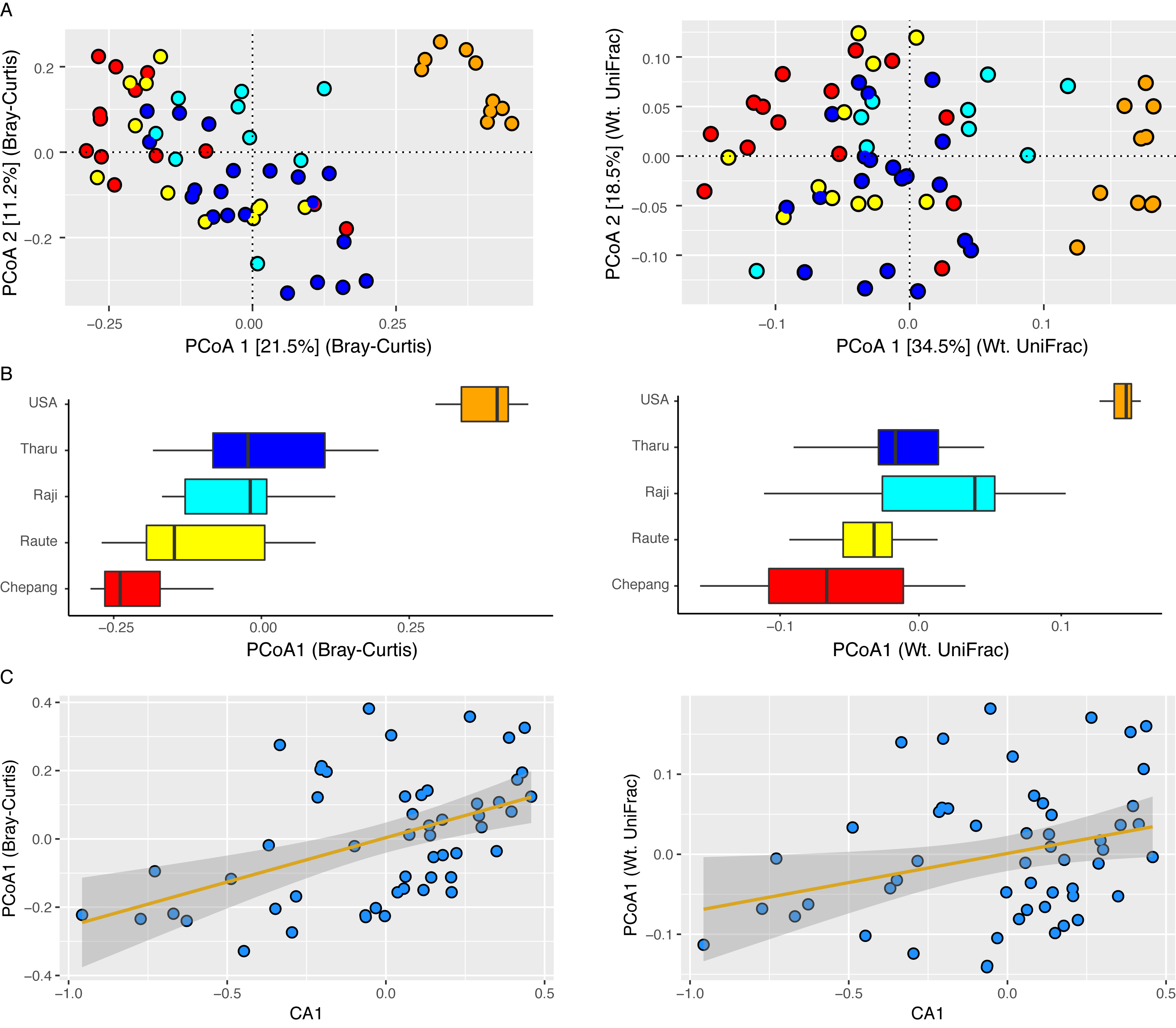

Supplement: S5 Fig — (A) Visualization using a PCoA of the Bray–Curtis (left) and weighted UniFrac distances (right). Each dot represents an individual, and colors indicate the populations. (B) Distribution of populations along the PCoA1 axis shows patterns of separation by lifestyles. Chepang foragers (red), Raute (yellow) and Raji (cyan), Tharu farmers (blue), and Americans (orange). (C) In both cases, PCoA1 was strongly correlated with CA1 obtained from the analysis of the survey data. Spearman’s Rho for Bray–Curtis and weighted UniFrac were 0.47 and 0.28, respectively (P < 0.05 for both, correlation test). Correlations between CA2 and PCoA1 were insignificant (P > 0.05 for both distances, correlation test). CA, correspondence analysis; PCoA, Principal Coordinate Analysis. (TIF) [file pbio.2005396.s005.tif]

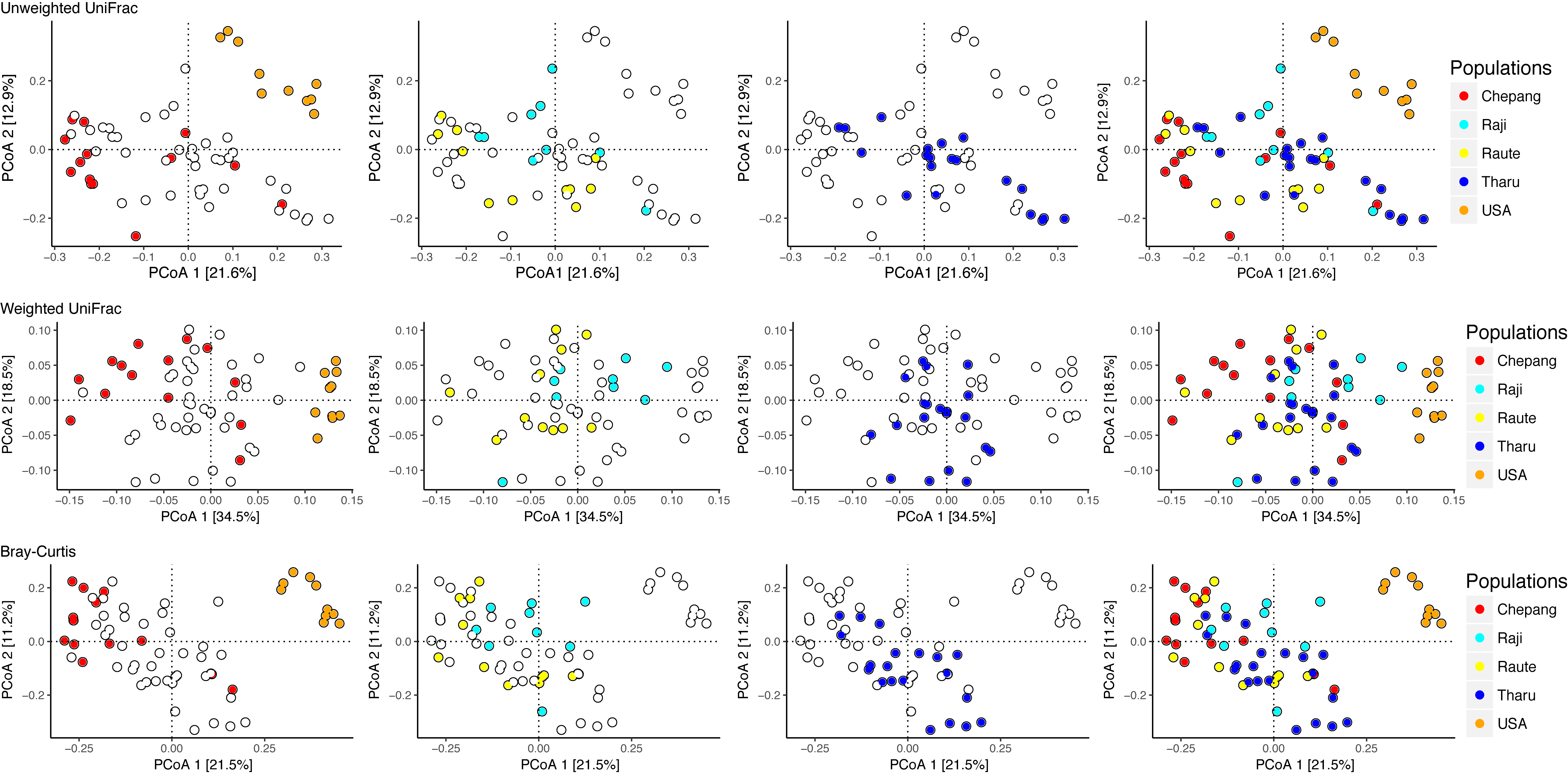

Supplement: S6 Fig — PCoA of the unweighted and weighted UniFrac distances (top and middle, respectively) and Bray–Curtis distance (bottom). All four plots on each row differ only in coloring of the dots to help visualize the distribution of individuals in each population. PCoA, Principal Coordinate Analysis. (TIF) [file pbio.2005396.s006.tif]

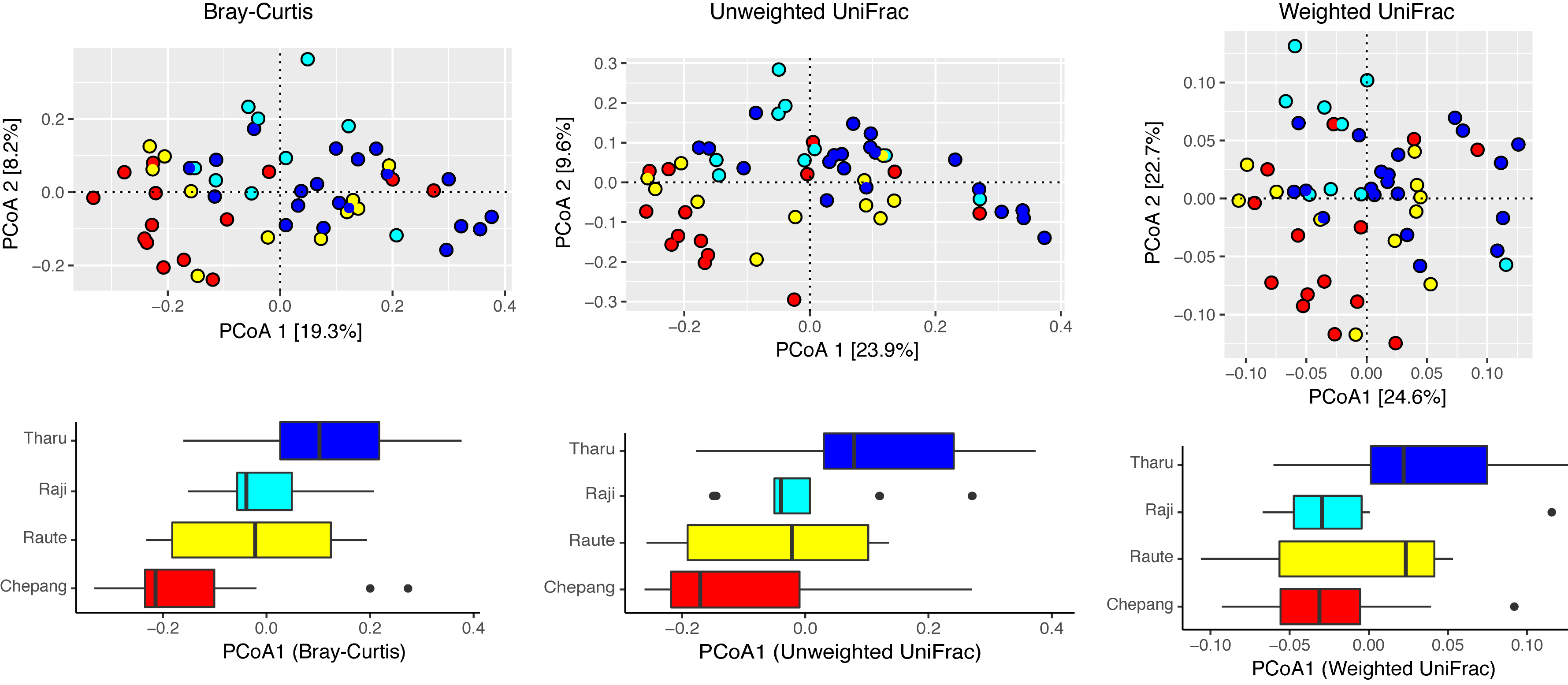

Supplement: S7 Fig — Columns show PCoA of the three distance matrices of the four Himalayan populations after removing Americans from the analysis. Top row shows the top two PCoA axes and variance explained. Significant differences in gut microbiome composition within Himalaya were observed for all three distances (P < 0.05, PERMANOVA). Bottom row shows the distribution of the Himalayan populations along the PCoA1 axis. The separation between Chepang foragers (red) and Tharu farmers (blue) is the strongest within Himalaya; the two transitioning Raute and Raji populations are intermediates. PCoA, Principal Coordinate Analysis; PERMANOVA, permutational multivariate analysis of variance. (TIF) [file pbio.2005396.s007.tif]

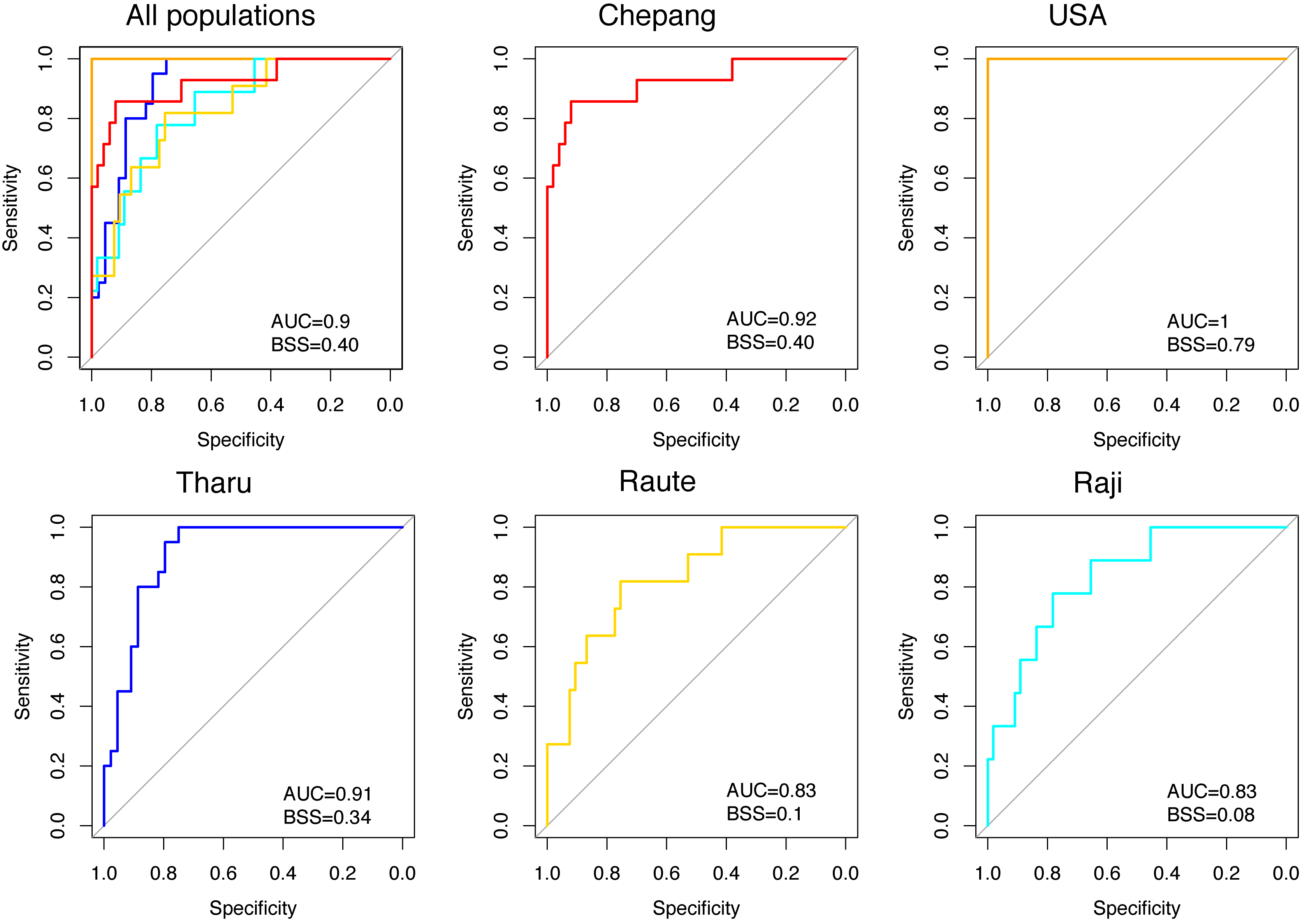

Supplement: S8 Fig — AUCs were computed to evaluate the performance of the RF model for each population. AUC for all populations was calculated by averaging the AUC for each population. A second metric, the BSS, was used to assess calibration of the RF model. A BSS = 0 means RF model performance is no different than random assignment, a negative BSS would suggest worse performance than random assignment, and BSS > 0 indicates that the RF model performed better than a random assignment. A BSS score of 1 would indicate a perfect calibration. The AUC and BSS values for the Chepang, the Americans, and the Tharu are >0.90 and >0.3, indicating that the RF model was able to accurately differentiate individuals from these populations based on their gut microbiome. Low BSS scores for Raute and Raji suggest that the RF model was unable to accurately distinguish individuals from these populations based on their gut microbiome, consistent with two similar populations in a transition state. AUC, area under the receiver operating characteristic curve; BSS, brier skill score; RF, random forest. (TIF) [file pbio.2005396.s008.tif]

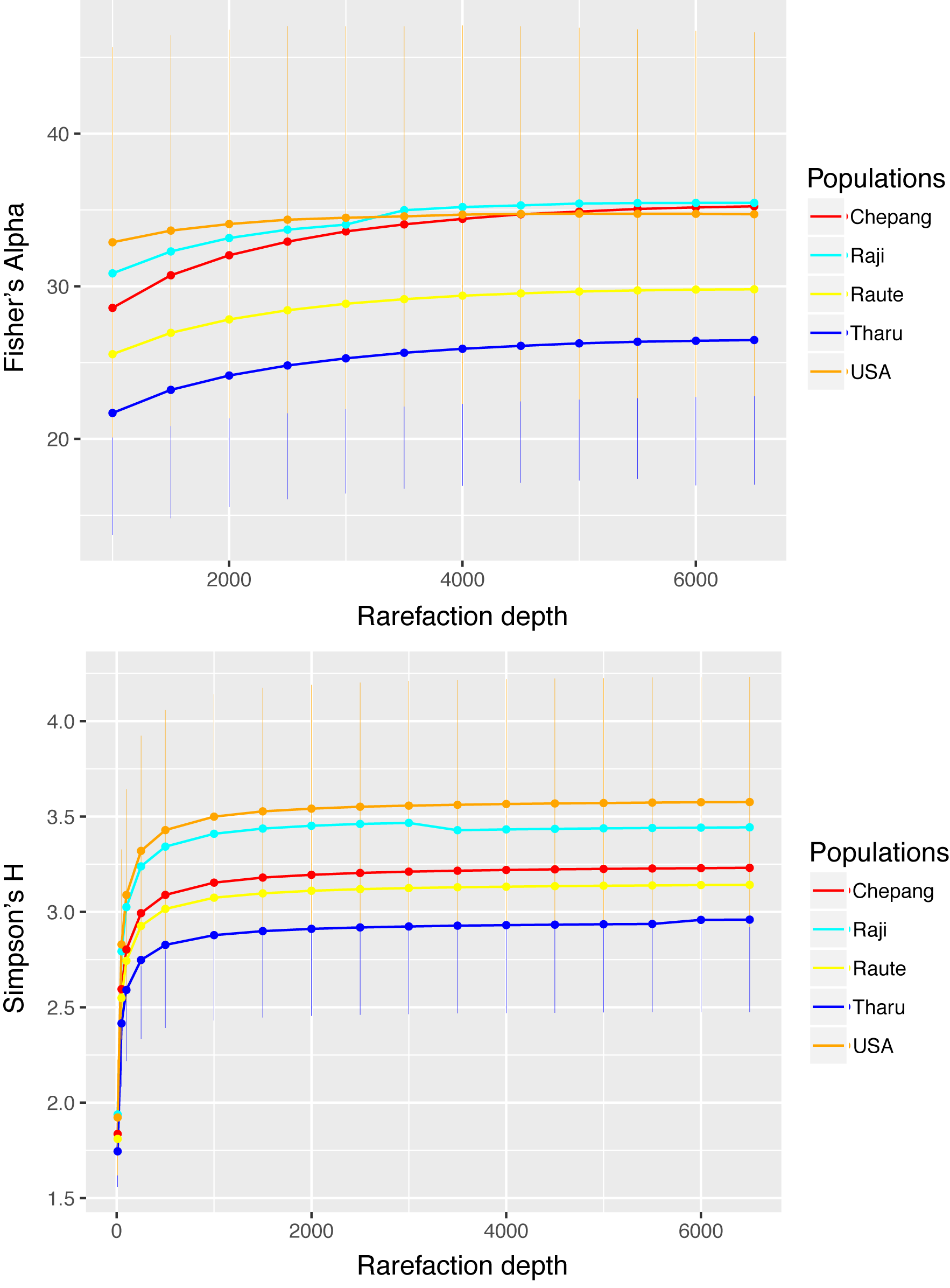

Supplement: S9 Fig — Rarefaction curves showing two additional commonly used measures of alpha diversity—Fisher’s alpha (top) and Simpson’s index (bottom) at different rarefaction depths (x-axes). No significant difference in Fisher’s alpha was detected across the five study populations. Simpson’s index was lower in the Tharu relative to the Americans, but none of the alpha diversity metrics showed significant differences between any of the four Himalayan populations. (TIF) [file pbio.2005396.s009.tif]

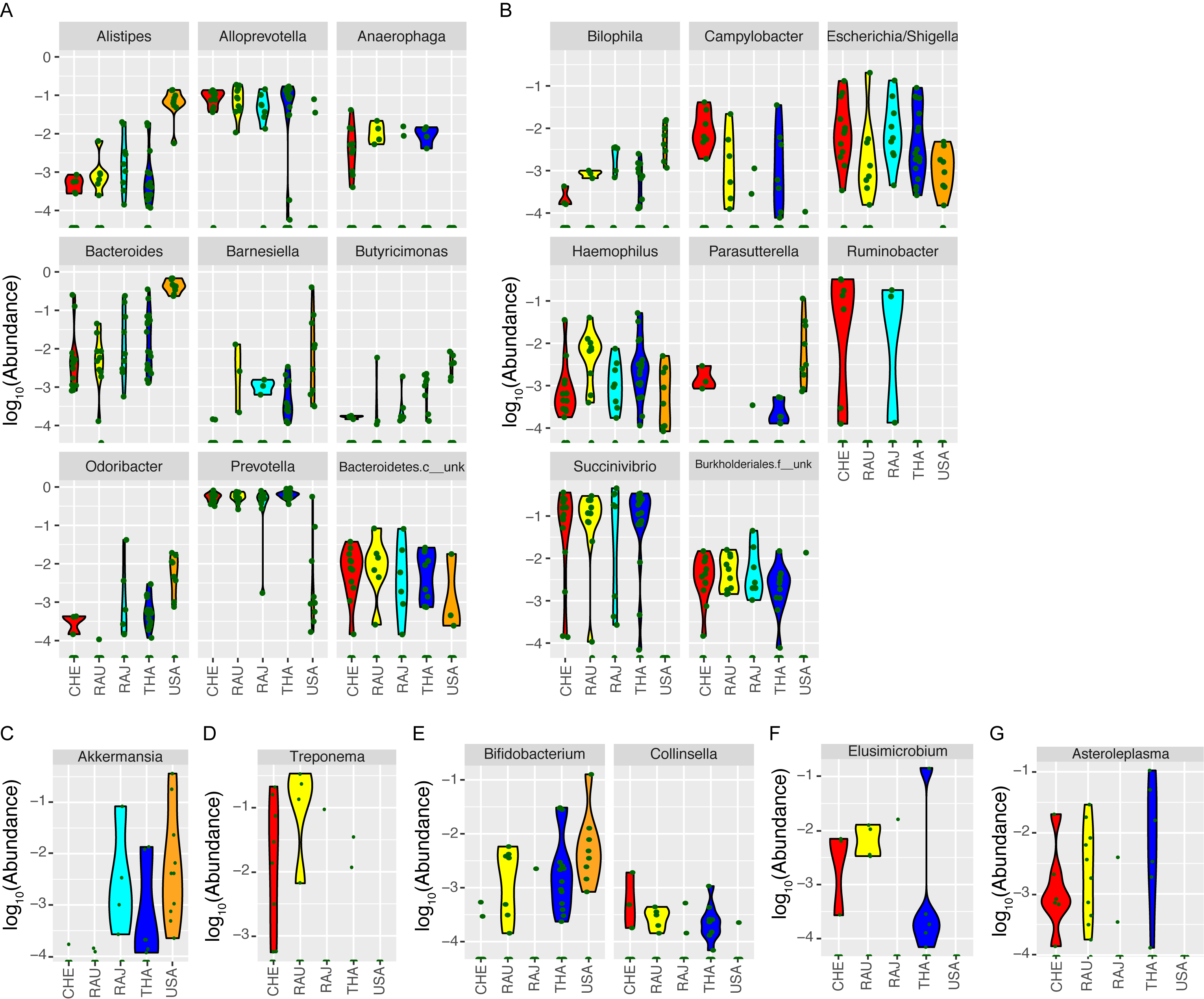

Supplement: S10 Fig — Each subplot shows abundance of an individual taxa in the five populations. Differentially abundant genera from Bacteroidetes (A), Proteobacteria (B), Verrucomicrobia (C), Spirocheates (D), Actinobacteria (E), Elusimicrobia (F), and Tenericutes (G). Labels with “c__unk” and “f__unk” indicate taxa with unknown class and family, respectively. (TIF) [file pbio.2005396.s010.tif]

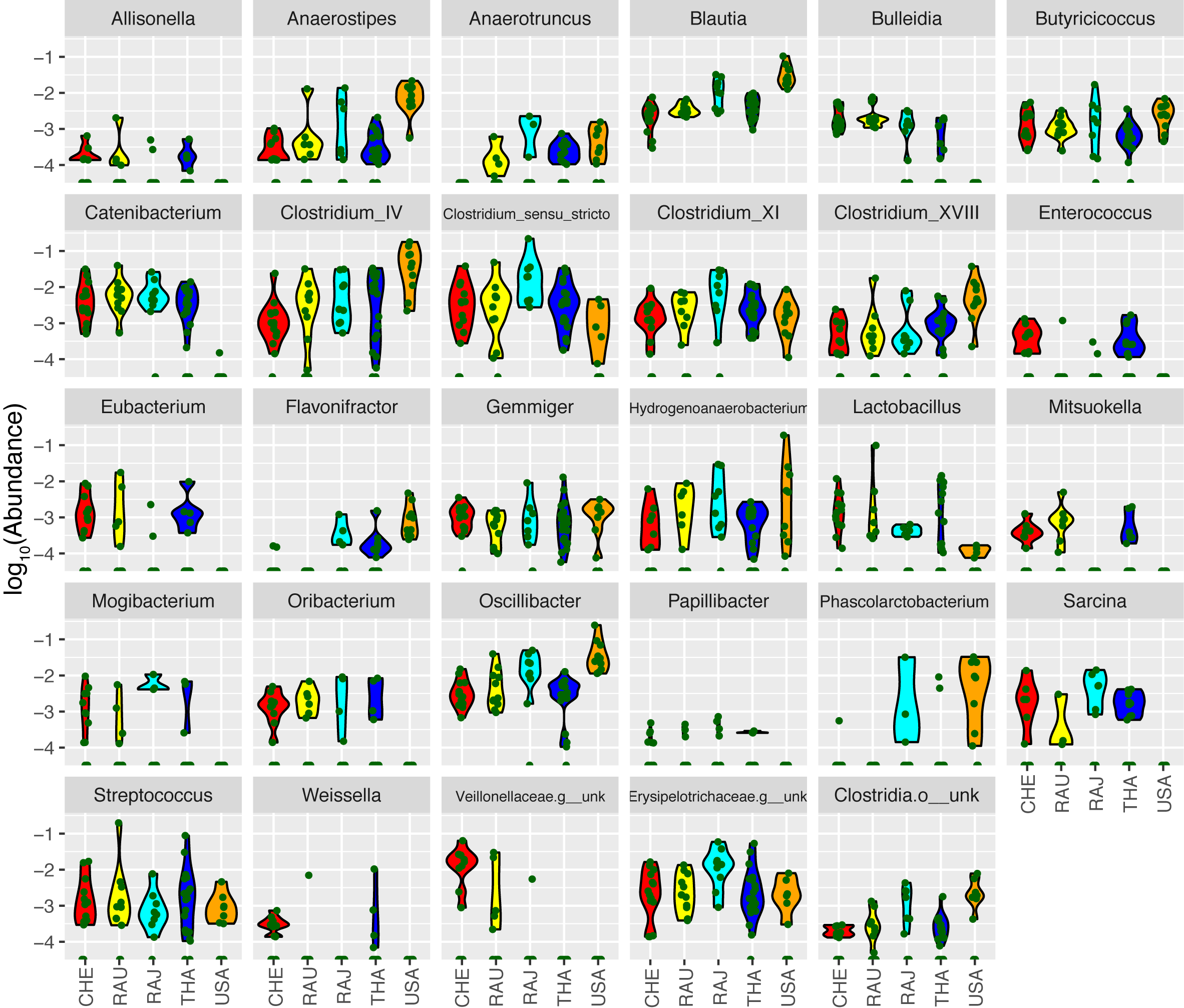

Supplement: S11 Fig — Several genera are significantly enriched in the rural Himalayan populations, and others are depleted. Labels with “g__unk” and “o__unk” indicate taxa with unknown genus and order, respectively. (TIF) [file pbio.2005396.s011.tif]

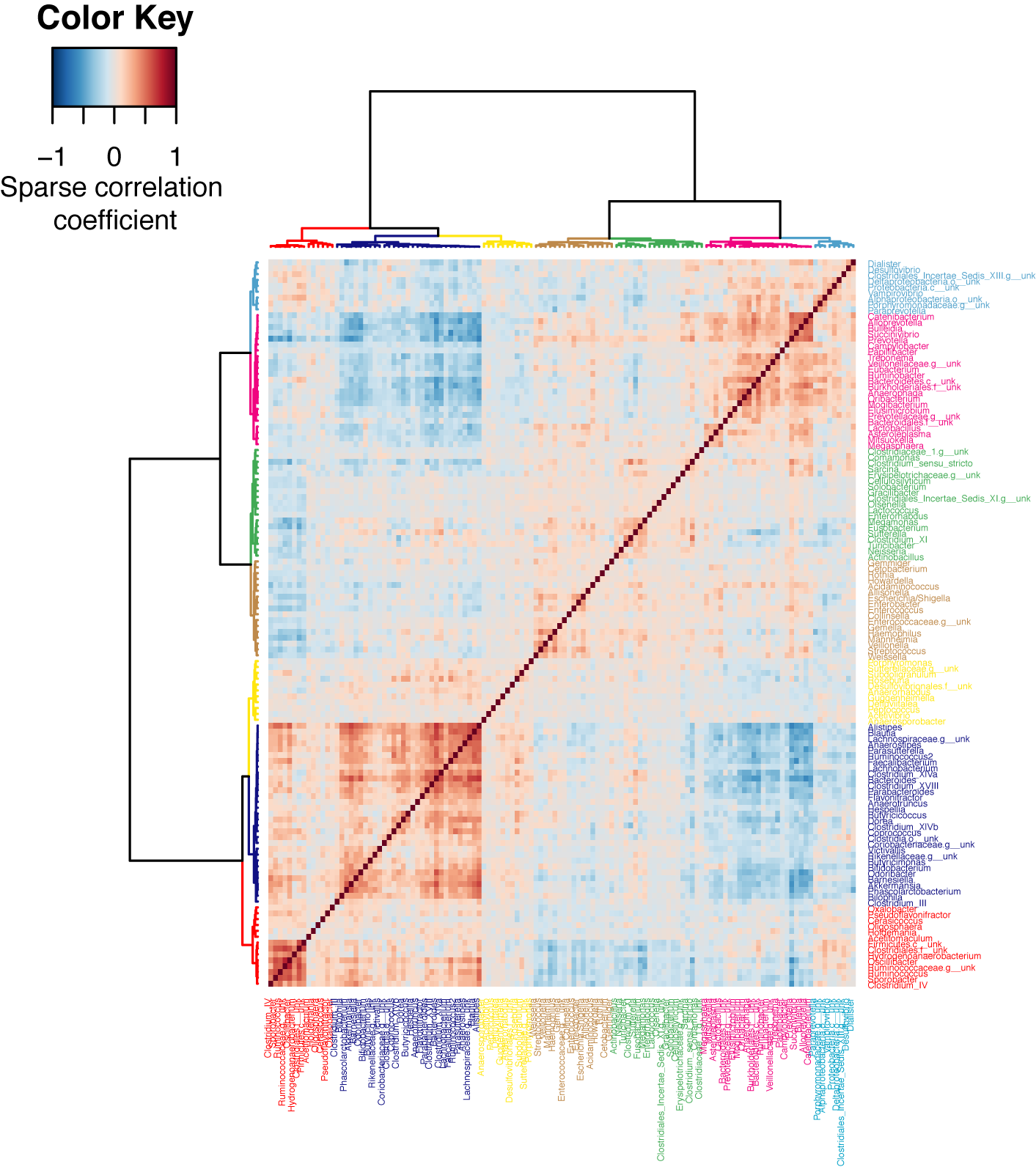

Supplement: S12 Fig — Compositionality robust correlations between bacterial genera across all samples (N = 64) were calculated using SparCC. Genera with less than 2 reads in at least 5% of samples (three individuals) were removed from this analysis. Ward’s hierarchical clustering performed using the correlation metric distance revealed bacterial CAGs. Dendrograms show that bacterial genera cluster into seven CAGs. Genera labels are colored based on their CAG memberships. Labels with “x__unk” indicate taxa with unknown classification level. c, class; CAG, coabundance group; f, family; g, genus; o, order; SparCC, Sparse Correlations for Compositional data. (TIF) [file pbio.2005396.s012.tif]

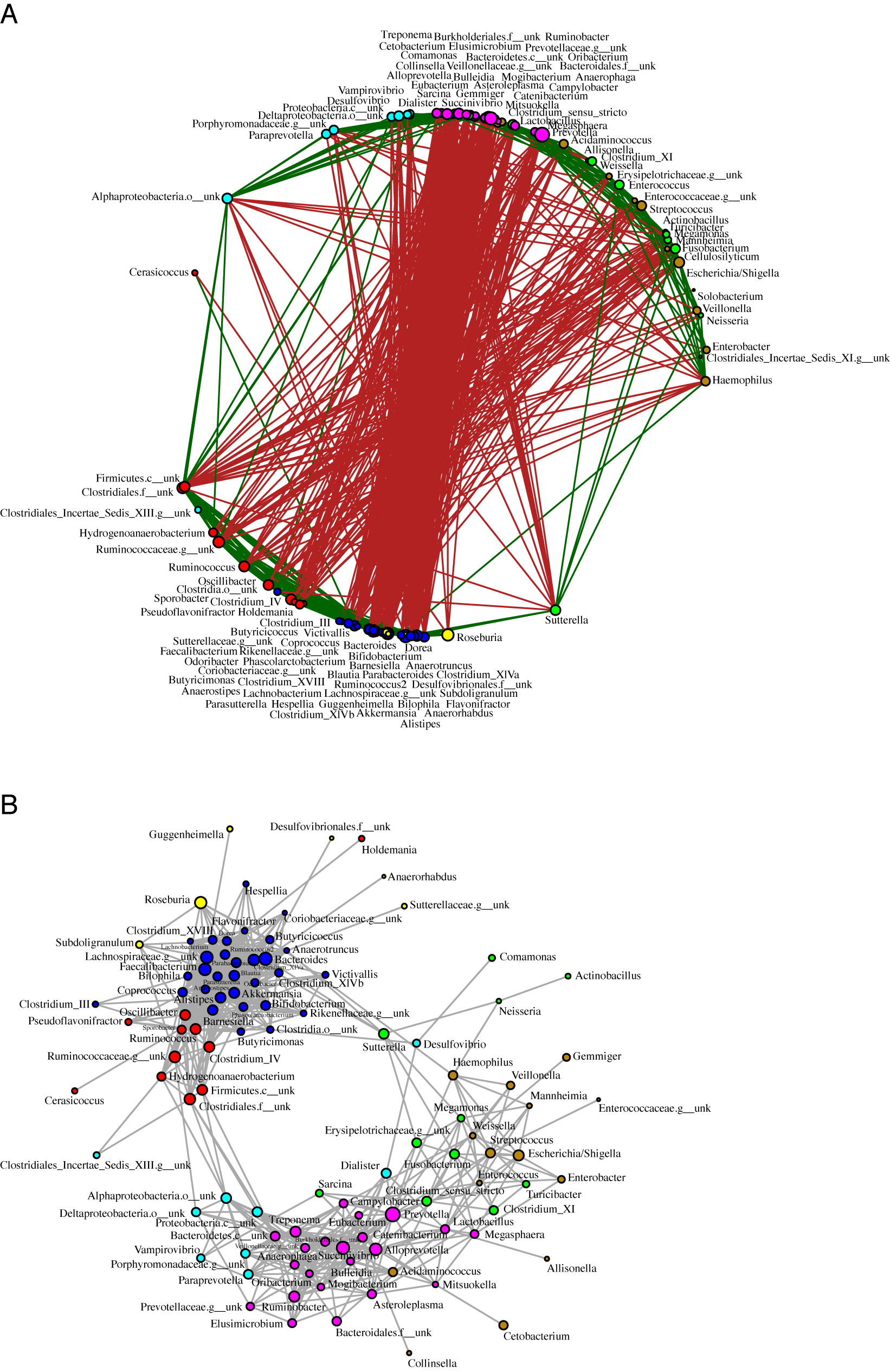

Supplement: S13 Fig — Significant associations between bacterial genera after adjusting for multiple testing (FDR adjusted P < 0.05) visualized using the Fruchterman–Reingold force–directed layout algorithm. Nodes (circles) represent bacterial genera, node colors represent the seven CAGs, and node sizes represent genus abundance. Edges represent the significant correlations between genera. (A) Edges colored in green and red show positive and negative correlations between the genera, respectively. Members of the red, blue, and yellow CAGs are mostly negatively correlated with the members of cyan, magenta, gold, and green CAGs. (B) Only the significant and positive correlations between bacterial genera are shown. Labels with “x__unk” indicate taxa with unknown classification level. c, class; CAG, coabundance group; f, family; FDR, false discovery rate; g, genus; o, order. (TIF) [file pbio.2005396.s013.tif]

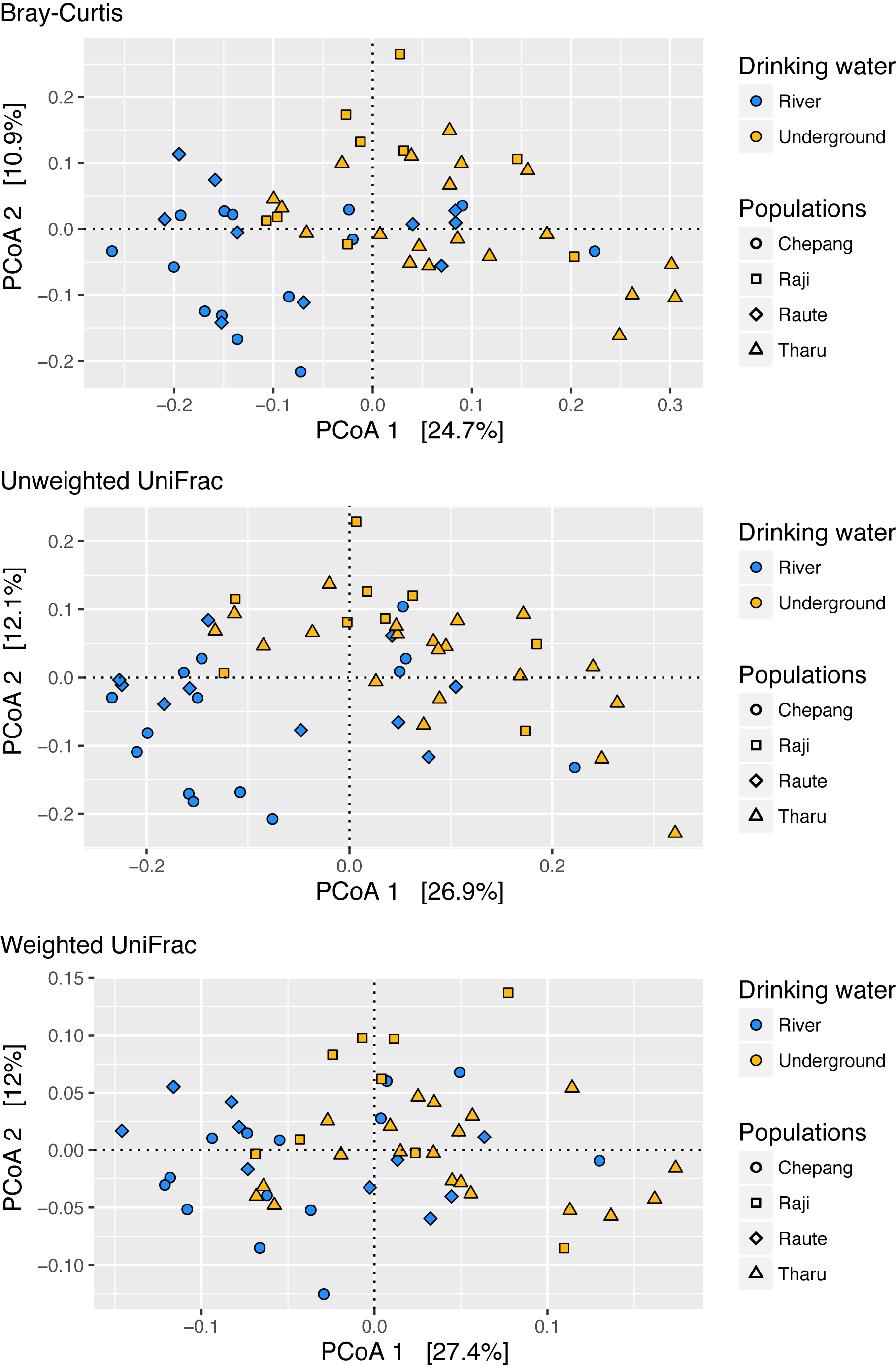

Supplement: S14 Fig — PCoA of the Bray–Curtis and weighted UniFrac distances. Each dot represents an individual, colors indicate the two drinking water sources, and shapes represent different populations. Gut microbiomes of the Chepang (circles) and Raute (diamonds) who drink water from rivers and streams vary significantly from those of the Raji (squares) and Tharu (triangles). Statistical significance was assessed using PERMANOVA using the 10 variables that differentiate their lifestyles (P value = 0.0001 for both distances). PCoA, Principal Coordinate Analysis; PERMANOVA, permutational multivariate analysis of variance. (TIF) [file pbio.2005396.s014.tif]

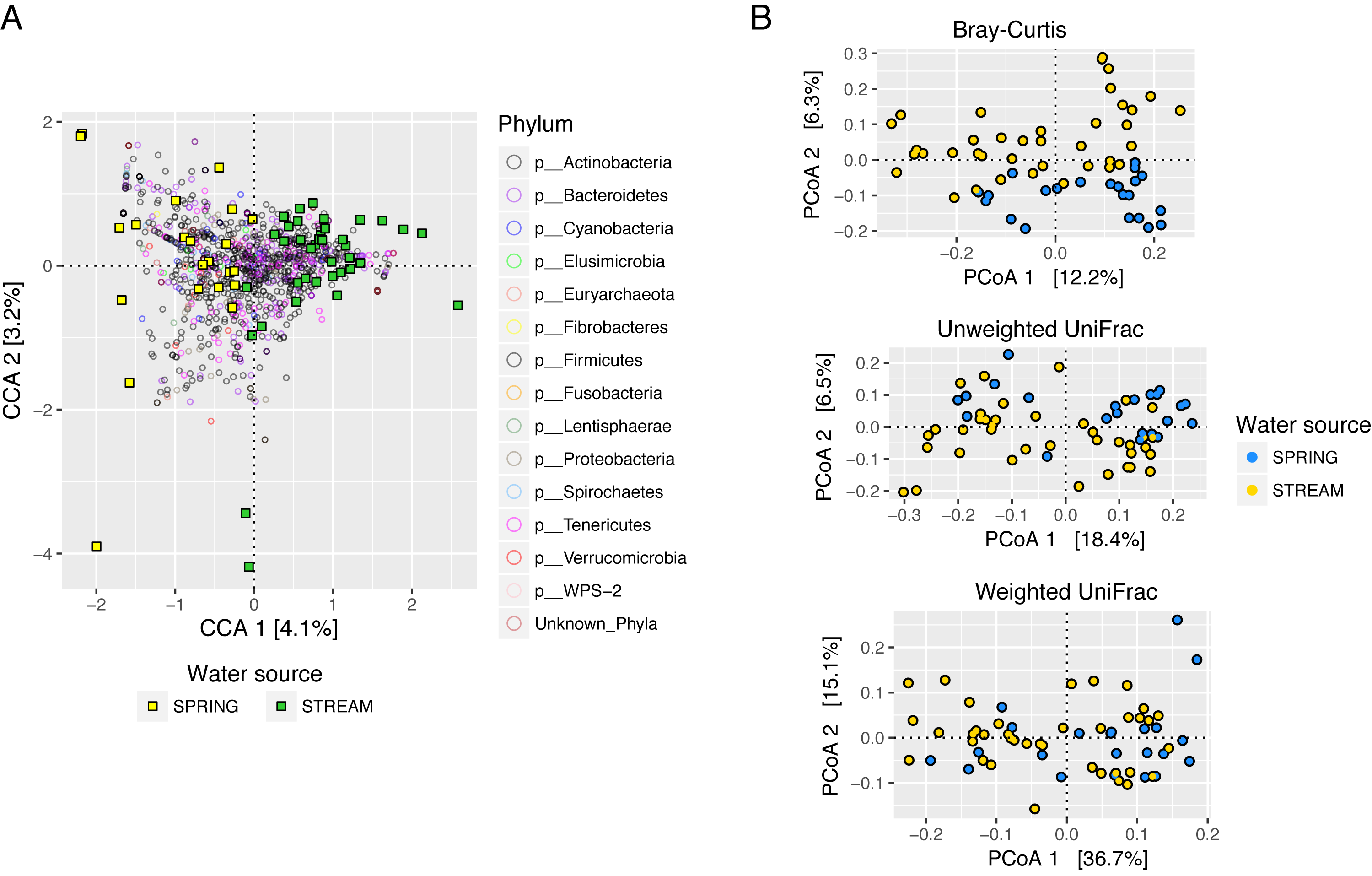

Supplement: S15 Fig — (A) CCA biplot showing separation between gut microbiome of Hadza from the Hukamako camp; all samples from late dry season. Individuals who use spring and stream water are shown in yellow and green squares, respectively. Circles represent bacterial RSVs, and the proportions of constrained variance explained by the two primary CCA axes are shown. (B) The two primary principal coordinate axes of Bray–Curtis, unweighted UniFrac, and weighted UniFrac distances in the Hukamako Hadza late dry season samples are shown along with the fraction of variance they explain. Each dot represents an individual, and colors indicate the two drinking water sources. Statistical significance was assessed using PERMANOVA (P value < 0.05 for all three distances). CCA; PERMANOVA, permutational multivariate analysis of variance; RSV, read sequence variant. (TIF) [file pbio.2005396.s015.tif]

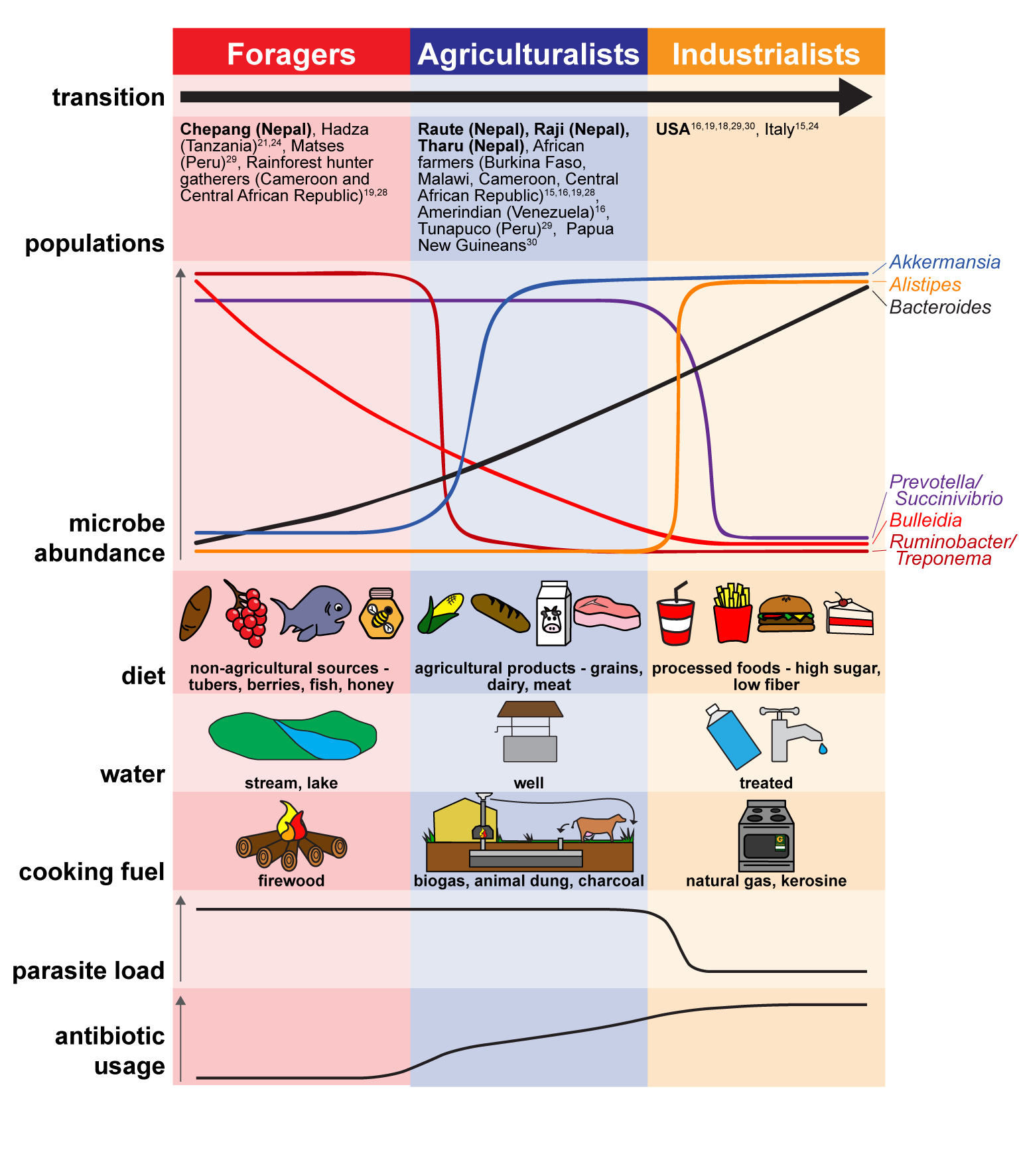

Supplement: S16 Fig — We propose that fluctuations in individual gut taxa show complex patterns as humans transition from one lifestyle to another. A few examples of bacterial taxa and their consistent patterns of changes in human populations around the world are shown. Certain genera such as Treponema and Ruminobacter that are characteristic of hunter–gatherers decline in agrarians and industrialists. In contrast, taxa such as Alistipes and Akkermansia increase in nonforagers. Genera such as Bacteroides show a gradual increase from foragers to industrialists and Bulleidia shows an opposite trend. Higher abundances of taxa such as Prevotella and Succinivibrio are characteristics of traditional lifestyles and are absent or rare in industrialists. Both dietary and environmental factors are likely to influence the gut microbiome. In this study, source of drinking water was strongly associated with gut microbiome composition. Other environmental factors such as parasite load and antibiotic usage also influence the gut microbiota. (TIF) [file pbio.2005396.s016.tif]
