# Supplementary material for: Gut microbiome transition across a lifestyle gradient in Himalaya
Source: PLoS Biol. 2018 Nov 15;16(11):e2005396. doi: 10.1371/journal.pbio.2005396 (PMC6237292; doi:10.1371/journal.pbio.2005396)
Supplement: S1 Alternative Language Abstract — (PDF) [file pbio.2005396.s026.pdf]

यस् अध्ययनको नेपालीमा सारांश

मानवीय आन्द्रामा धेरै प्रकारका सुक्ष्म जीवाणुहरुले बास गरेका हुन्छन्। यी जीवाणुहरुको उपस्थिती मानिसको स्वास्थ्यका लागि निक्कै महत्वपूर्ण हुन्छ भन्ने बैज्ञानिक मान्यता छ। औद्योगिक राष्ट्रमा बसोबास गर्ने मानिसको आन्द्रामा रहेका जीवाणुहरुको रचना पारम्पारिक जीवनशैली व्यतीत गर्ने मानवहरुको भन्दा धेरै भिन्न हुन्छ। तर यी भिन्नताहरुमा आणुवंशिक, भौगोलिक, र जीवनशैलीले के कस्ता योगदान गर्छन् भन्ने थाहा पाउन सकिएको छैन।

२००,००० बर्ष अघी आफ्रिकामा उत्पत्ती भए पछी मानव ईतिहासका हज्जारौं बर्ष मानिसले जंगलबाट खानेकुरा बटुलेर आफ्नो जीवन गुजरा गर्‍यो। १०,००० बर्ष अगाडि मानिसले कृषि गर्न सिक्यो र त्यसपछी भ्रमणशील जीवनशैली त्यागेर एकै ठाउँमा बसोबास गर्न थाल्यो। यसैगरी १००-२०० बर्ष अघि मानिसहरुले औद्योघिक जीवन अपनाउन थालेका छन्। जङलमा निर्भर हुने अवस्थाबाट अन्य प्रकारका जीवनशैली अपनाउन थालेपछी मानिसको जीवनशैलीमा आएका परिवर्तनहरुले मानिसको आन्द्रामा हुने सुक्ष्म जीवाणुहरुमा के कस्ता असर परेका छन् थाहा भाईसकेको छैन। त्यसैले हामीले पारम्पारिक जीवनशैलीहरुको परिवर्तन साथै आन्द्राको जीवाणुहरुको रचनामा के कस्ता उतार-चढावहरु आउँदा रहेछन भन्ने कुराको अनुसन्धान गर्न हिमालयमा बस्ने चार जनजातिहरुको आन्द्राका ब्याक्टेरियाहरुको अध्ययन गरेका छौं। यी चार हिमाली समुदायहरु - चेपाँग, राउटे, राजी, र थारु - कृषि पेशामा निर्भर हुनुभन्दाअघि अर्धभ्रमणशील जीवनशैली व्यतित गर्दै जङलमा खानेकुराहरुको शिकार र संकलन गर्दथे। थारुहरु २५०-३०० बर्ष अघि तथा राउटे र राजी ३०-४० बर्ष अघि कृषि पेशामा संलग्न हुन थालेका हुन् भने चेपाँङहरू अझैपनी जङलमा निर्भर हुने हुनाले उनिहरुले आफ्नो परम्परागत जीवनशैलीका धेरै पक्षहरु अझैसम्मपनि कायमै राखेका छन्।

यस् अध्ययनमा हामीले मानिसको अहार तथा पर्यावरणका विभिन्न कारणहरुले कसरी उनिहरुका आन्द्राका जीवाणुहरुलाई असर गर्छन् भन्ने कुराको मुल्याङ्कन गर्दा चेपाँङ र थारुहरुको जीवनशैलीमा पाइएका अन्तरहरुले उनिहरुको आन्द्राका जीवाणुहरुको रचनालाई निक्कै प्रभाव पार्नसक्ने पाएका छौं। हिमालयमा वसोवास गर्दै आएका यी चारै समुदायहरुको आन्द्राका जीवाणुहरु अमेरिकनहरुको भन्दा भिन्नै पाइएको छ जसले के जनाउँछ भने औद्योगिकरणले आन्द्राका जीवणुहरुमा थप भिन्नताहरुको श्रीजना गर्नसक्छ। हिमालयमा अर्धभ्रमणशील जीवनयापन गर्दै आएका चेपाँङहरुमा संसारका अन्य पारम्पारिक शिकारी जीवनशैली व्यतित गर्ने मानव समुदायहरुमा पाइने जस्ता जीवाणुहरु अधिकमात्रामा पाइएको छ। विपरीततः कृषि पेशामा पूर्ण रूपमा परिवर्तित भईसकेको समुदायको आन्द्रामा पाइने जीवाणुहरु भने औद्योगिक राष्ट्र अमेरिकामा बस्ने मानव समुदायहरुसँग बढी मिल्दो जुल्दो पाइएको छ। राउटे र राजीका आन्द्राका जीवाणुहरु चेपाँङ र थारुहरुको बीचको अवस्थामा छन् जसले के देखाउँछ भने परम्परागत अवस्थाबाट आन्द्राको जीवाणुहरुको बिचलन हुन धेरै समय लाग्दैन। यसरी आन्द्राको जीवाणुहरुको रचनामा परम्परागत अवस्थाबाट बिचलन हुनुमा खाने पानी तथा खाना पकाउने ईन्धन जस्ता पर्यावरणसँग सम्बन्धित विभिन्न कारकहरुको पनि भूमिका हुनसक्ने देखिएको छ। यसरी विभिन्न किसिमका रहनसहन भएका मानव समुदायहरुका जीवाणुहरुको रचनामा स्पष्ट अन्तरहरु देखिएता पनि उनिहरुका आन्द्रामा भएका जीवाणुहरुका सन्ख्यामा भने उत्ती फरक देखिएन।

समग्रमा, एउटै भौगोलिक क्षेत्रमा उस्ता उस्तै जीवनशैली व्यतीत गर्दै आएका यी चार नेपाली आदिवासी समुदायहरुमा भएको यस् अध्ययनले मानिसको जीवनशैलीले आन्द्रामा भएका जीवाणुहरुको रचनामा निक्कै प्रभाव पार्छन भन्ने स्पष्टरुपमा देखाएको छ र साथै जीवनशैलीको परिवर्तनले आन्द्राका जीवाणुहरुमा ल्याउने अन्तरहरुले मानिसको स्वास्थ्यमा के कस्ता प्रभाव पार्न सक्छन भन्ने चुनौतिपूर्ण कदमहरु चाल्न अत्यन्त आवश्यक भएको देखाएको छ।
